# Amyloplasts are necessary for full gravitropism in thallus of *Marchantia polymorpha*

**DOI:** 10.1093/jxb/eraf375

**Published:** 2025-08-19

**Authors:** Mimi Hashimoto-Sugimoto, Takuya Norizuki, Shoji Segami, Yusaku Ohta, Noriyuki Suetsugu, Takashi Ueda, Miyo Terao Morita

**Affiliations:** Graduate School of Bioagricultural Sciences, Nagoya University, Nagoya 464-8601, Japan; Laboratory of Molecular Membrane Biology, Institute for Molecular and Cellular Regulation, Gunma University, Maebashi 371-8512, Japan; Division of Cellular Dynamics, National Institute for Basic Biology, Okazaki 444-8585, Japan; Graduate School of Bioagricultural Sciences, Nagoya University, Nagoya 464-8601, Japan; Division of Evolutionary Biology, National Institute for Basic Biology, Okazaki 444-8585, Japan; Course for Basic Biology, The Graduate Institute for Advanced Studies, SOKENDAI, Hayama 240-0115, Japan; Course for Basic Biology, The Graduate Institute for Advanced Studies, SOKENDAI, Hayama 240-0115, Japan; Bioimage Informatics Group, Exploratory Research Center on Life and Living Systems (ExCELLS), National Institutes of Natural Sciences, Okazaki 444-8787, Japan; Interdisciplinary Research Unit, National Institute for Basic Biology, National Institutes of Natural Sciences, Okazaki 444-8787, Japan; Department of Life Sciences, Graduate School of Arts and Sciences, The University of Tokyo, Tokyo 153-8902, Japan; Division of Cellular Dynamics, National Institute for Basic Biology, Okazaki 444-8585, Japan; Course for Basic Biology, The Graduate Institute for Advanced Studies, SOKENDAI, Hayama 240-0115, Japan; Course for Basic Biology, The Graduate Institute for Advanced Studies, SOKENDAI, Hayama 240-0115, Japan; Division of Plant Environmental Responses, National Institute for Basic Biology, Okazaki 444-8585, Japan; Cardiff University, UK

**Keywords:** Amyloplast, gravitropism, *Marchantia polymorpha*, starch, statolith, thallus

## Abstract

Gravitropism is a plant response to gravity that directs organ growth and development, playing a key role in the adaptation of land plants. While its molecular basis has been extensively studied in flowering plants, much less is known about this process in other plant lineages. Here, we investigated the gravitropic response of the liverwort *Marchantia polymorpha*, a model for early land plant evolution. In darkness, the thallus tips extended upward, forming several straight, narrow structures whose growth direction was consistently opposite to gravity and disrupted by clinostat treatment. These structures contained amyloplasts in parenchymatous cells, and their sedimentation preceded gravitropic curvature, suggesting a role as statoliths. Amyloplast sedimentation started near the tip and slowed with distance, and in more distal regions, both the size and the number of amyloplasts decreased. In starchless mutants (Mp*pgm1* and Mp*aps1*), the narrow structures displayed abnormal growth directions, although they still tended to elongate upward. These results indicate that while amyloplasts are required for proper gravitropism, *M. polymorpha* retains the ability to sense gravity even without well-developed amyloplasts. Our findings suggest that land plants use amyloplasts as statoliths but also possess amyloplast-independent mechanisms for gravitropic sensing.

## Graphical Abstract

**Graphical Abstract:**
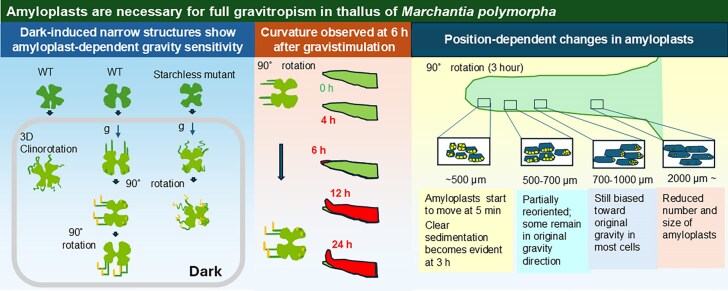

## Teaser

In *Marchantia polymorpha*, amyloplasts function as statoliths in parenchymatous storage cells and play an important role in gravitropism; however, gravitropism is not completely abolished in the absence of starch granules.

## Introduction

Plants are essentially sessile organisms and can sense various environmental stimuli and then adjust their growth direction to a position more suitable for water absorption, photosynthesis, and reproduction. Gravity and light are the most important directional environmental stimuli controlling growth direction (Knight, 1806; Hangarter, 1997; Kiss, 2000). Different from other environmental stimuli, gravity is continuously present, so plants can use this reliable indicator of their direction even in dark conditions. Gravitropism in plants is the development or growth of plant organs in a specific direction in response to gravity and has four sequential steps: gravity perception, signal formation, signal transduction, and differential growth of the upper and lower tissues of the responding organ (Tasaka *et al*., 1999; Morita and Tasaka, 2004; Morita, 2010).

In various stem and root tissues of flowering plants, gravity-sensing organelles, or gravity susceptors, are believed to sediment within specialized cells (statocytes) involved in gravity perception (Haberlandt, 1900; Němec̀, 1900). Extensive research has supported the starch statolith theory of gravity perception in gravitropism (Sack, 1991; Konings, 1995; Blancaflor and Masson, 2003). Physiological and genetic studies in Arabidopsis have provided evidence that amyloplasts, starch-filled plastids, are essential for full gravitropic sensitivity in both roots and shoots. For example, the *pgm* mutant, which lacks plastidial phosphoglucomutase activity and is deficient in starch, exhibits a markedly reduced gravitropic response (Caspar and Pickard, 1989; Kiss *et al.*, 1989, 1997). Similarly, other starchless or starch-reduced mutants that contain immature amyloplasts show reduced gravitropism in both the shoot and the root (Kiss *et al.*, 1989, 1996, 1997; Weise and Kiss, 1999). Although direct evidence is limited, it has been proposed that the mass of amyloplasts, which depends on their starch content, may affect the extent of the gravitropic response (Kiss *et al*., 1996).　

As statoliths in non-angiosperm plants, vesicles filled with aggregates of barium sulfate crystals have been reported in the alga *Chara* spp. living in water (Sievers *et al*., 1991; Braun and Sievers, 1993; Hodick *et al*., 1998). Much of the early work on the effects of gravity on bryophytes was summarized by Petschow (1933). He found that some mosses and liverworts had intracellularly sedimented starch granules in their thalli and grew upward in the dark. Furthermore, amyloplast sedimentation and upward curvature also appeared in single cell protonemata of mosses, suggesting a correlation between the perception of gravity and settled statoliths (Petschow, 1933; Sack *et al*., 2001). However, recent work revealed that rhizoids of the model moss *Physcomitrella patens* (*Physcomitrium patens*) exhibit gravitropism but did not find any detectable starch granules (Zhang *et al*., 2019). It remains unclear whether the starch granules are required as gravitropic signaling factors in bryophytes, partly because molecular genetic analysis was poorly developed. *Marchantia polymorpha*, a liverwort, has recently been established as a model plant species with available genomic information (Bowman *et al*., 2017; Bowman, 2022) and various tools for molecular genetic analysis such as CRISPR/Cas9-based genome editing (Sugano *et al*., 2018; Kohchi *et al*., 2021). In this study, we focused on the gravity responsiveness of thalli of *M. polymorpha* and investigated its basic characteristics. In addition, genetic and physiological analyses were conducted to understand the role of starch grains in the response.

## Materials and methods

### Plant material and growth conditions

*Marchantia polymorpha* Takaragaike-1 (Tak-1) was used as a wild type and genetic background for transgenic lines. To observe MpPGM1-Citrine, transgenic plants expressing *_pro_*Mp*EF1α:*Mp*PGM1-Citrine* (Norizuki *et al*., 2023) were used. Gemmae of *M. polymorpha* plants were horizontally grown on half-strength Gamborg B5 medium supplemented with 1% (w/v) sucrose (pH 5.5, 1% agar) (Gamborg *et al*., 1968) at 22 °C under continuous light. To generate narrow structures from the original thallus, plates of the light-grown thalli were placed vertically in darkness. After some elongated portions of the thallus emerged, the plates with plants on them were turned 90°. The experiment to determine if a part of the thallus buried in the soil would come out of the ground was conducted as follows. Two-week-old light-grown thalli were covered with the soil of a 1:1 (v/v) mixture of vermiculite and Metromix 350 (Scotts-Sierra Horticultural Products, USA) 1 cm deep and then grown with 5000-fold diluted Hyponex solution (Hyponex, Japan) applied as nutrient for 5 weeks at 22 °C under continuous light.

### Identification of *Marchantia* phosphoglucomutases and small and large subunits of ADP-glucose pyrophosphorylases

Amino acid sequences of Mp*PGM1* (Mp4g13750.1/Mapoly0202s0014.1), Mp*PGM2* (Mp5g10560.1/Mapoly0048s0016.1), Mp*APS1* (Mp1g15530.1/Mapoly0033s0108.1), Mp*APL1* (Mp2g11530.1/Mapoly0023s0119.1), Mp*APL2* (Mp4g20740.1/Mapoly0101s0020.1), and Mp*APL3* (Mp4g10120.1/Mapoly0132s0055.1) were obtained from *M. polymorpha* genome version 5.1 (Montgomery *et al*., 2020) in MarpolBase (http://marchantia.info/) using these proteins of Arabidopsis as queries. The full amino acid sequences are provided in Supplementary Tables S1 and S2. A domain search was performed using SMART (http://smart.embl-heidelberg.de/) (Letunic and Bork, 2018; Letunic *et al*., 2021). Transit peptides of MpPGM1 and MpAPS1 were deduced using ChloroP 1.1 Server (https://services.healthtech.dtu.dk/services/ChloroP-1.1/) (Emanuelsson, *et al*., 1999). We followed the nomenclature of Bowman *et al*. (2016) for genes, proteins, and mutants of *M. polymorpha*.

### Phylogenetic analysis of phosphoglucomutases and small and large subunits of ADP-glucose pyrophosphorylases

Phylogenetic analyses of phosphoglucomutases (PGMs), small subunits of ADP-glucose pyrophosphorylases (APSs), and large subunits of ADP-glucose pyrophosphorylases (APLs) were according to Norizuki *et al*. (2019). As a substitution model, we used the WAG+G+I (for PGM) or the LG+G+I+F (for APS and APL) model, which was selected by Smart Model Selection in PhyML (Lefort *et al*., 2017). Bootstrap analysis was performed by resampling 1000 sets. The sequences used in the phylogenetic analysis are included in Supplementary Table S1.

### Construction and transformation

To construct *pro*Mp*EF1α:*Mp*APS1-Citrine*, open reading frames of Mp*APS1* were amplified by PCR from cDNA obtained from thalli of *M. polymorpha* accession Tak-1 using the oligonucleotides MpApS1_Fw and MpApS1_Rv, and the amplified product was subcloned into pENTR^TM^/D-TOPO (Thermo Fisher Scientific, USA) according to the manufacturer’s instructions. The resultant sequence was then introduced into pMpGWB308 (Ishizaki *et al*., 2015) using the Gateway LR Clonase^TM^ II Enzyme Mix (Thermo Fisher Scientific) according to the manufacturer’s instructions. To construct CRISPR/Cas9 vectors, two complementary oligonucleotides in the sequences of Mp*PGM1* (MpPGM1_gDNA_Fw and MpPGM1_gDNA_Rv) and Mp*APS1* (MpAPS1_gDNA_Fw and MpAPS1_gDNA_Rv) were synthesized and annealed, and the resulting double-stranded fragments were subcloned at the *Bsa*I site of the pMpGE_En03 vector (Sugano *et al*., 2018) using the DNA ligation kit Ver.2.1 (Takara Bio, Japan) according to the manufacturer’s instructions. The resultant guide RNA cassette flanked by the *att*L1 and *att*L2 sequences in pMpGE_En03 was then introduced into the pMpGE010 vector (Sugano *et al*., 2018) using the Gateway LR Clonase^TM^ II Enzyme Mix. The primer sequences used in this study are listed in Supplementary Table S3. The transformation was performed as previously described (Kubota *et al*., 2013). Transformants were selected on plates containing 10 mg l^−1^ hygromycin B and 250 mg l^−1^ cefotaxime for the pMpGE010 vectors and 0.5 μM chlorsulfuron and 250 mg l^−1^ cefotaxime for the pMpGWB308 vector.

### Genotyping

For the genotyping of mutants generated by CRISPR/Cas9, genomic DNA was extracted from thalli with an extraction buffer [1 M KCl, 100 mM Tris–HCl (pH 9.5), and 10 mM EDTA]. Genome regions of Mp*PGM1* and Mp*APS1* were amplified by PCR using KOD FX Neo (TOYOBO, Japan) using the oligonucleotides MpAPS1_GT1_Fw, MpApS1_Rv, MpPGM1_GT1_Fw, and MpPGM1_GT2_Rv, and mutations in these genome fragments were analysed by direct sequencing using the oligonucleotides MpAPS1_seq1_Fw and MpPGM1_seq1_Fw.

### Clinostat rotation

Thalli were grown on a plate in the light for 2 weeks. The plate was covered with aluminum foil to culture thalli in darkness and equipped with a 3D-Clinostat for 2 weeks (Plant Gravity Response Research Equipment, Kitagawa, Japan).

### Time-lapse images

As described above, thalli grown in the light for 2 weeks were placed on vertically held plates in the dark for 2 weeks to form the narrow structures. The plates were then turned 90° to a horizontal position. The development of narrow structures was recorded continuously in darkness with a time-lapsed video image recording camera (Brinno TC200 Pro; Brinno, Taiwan) with a low destruction lens (VS-LD4; VS Technology, Japan), using infrared radiation with a peak emission wavelength of 950 nm as the monitoring light (KB850-7224LE KS-602983; KOSU system, Japan).

### Lugol’s iodine staining

Thalli with protruding narrow structures were fixed in 80% (v/v) ethanol for 30 min under vacuum at room temperature. The fixed tissues were washed twice with water and stained in 0.05 mol l^−1^ iodide solution (Wako). This method did not remove the weak dark pigment (Supplementary Fig. S1A–C), so ClearSee solution was used before iodine treatment to more clearly compare staining differences between starch-deficient mutants and the wild type (Kurihara *et al*., 2015; Segami *et al*., 2018). Samples were fixed in 4% (w/v) paraformaldehyde in phosphate-buffered saline (PBS) for 30 min under vacuum at room temperature. The fixed tissues were washed twice for 1 min in PBS and cleared with ClearSee version1 [10% (w/v) xylitol, 15% (w/v) sodium deoxycholate, and 25% (w/v) urea]. Sodium deoxycholate has a weak decolorizing activity for iodine staining, so the samples were transferred to 10% (w/v) xylitol and 25% (w/v) urea. Finally, the tissues were stained in 0.05 mol l^−1^ iodide solution. Sample images were obtained by a digital microscope (Olympus DSX510, Olympus, Japan).

### Modified pseudo-Schiff–propidium iodide staining

Narrow structures of thalli were fixed in fixative [50% (v/v) methanol and 10% (v/v) acetic acid] for at least 16 h. The fixed tissue was rinsed with water and incubated in 1% (v/v) periodic acid at room temperature for 30 min. The tissue was rinsed again with water, and incubated in Schiff reagent with propidium iodide (100 mM sodium metabisulphite and 0.15 M HCl; propidium iodide to a final concentration of 100 mg ml^−1^ was freshly added) for 5 d in the dark. The samples were washed twice in water and then transferred onto microscope slides. Samples were cleared using a solution containing 80 g of chloral hydrate dissolved in 30 ml.

### Microscopy

Bright-field images of Lugol-stained samples were obtained using a digital microscope (DSX110; Olympus). For confocal microscopic observation of thallus cells expressing MpPGM1-Citrine or MpAPS1-Citrine, 5-day-old thalli were observed with an LSM780 confocal microscope (Carl Zeiss) equipped with an oil immersion lens (×63, numerical aperture=1.4) as described previously (Norizuki *et al*., 2023). Confocal laser scanning microscopy observations for modified pseudo-Schiff–propidium iodide (mPS-PI)-stained samples were conducted with an upright FV1000 confocal laser scanning microscope (Olympus). The excitation wavelength was 559 nm and the transmission range for emission was 655–755 nm. The images were obtained using Z-stacks (consecutive 5 µm thick optical slices) with Olympus FluoView software. Optimal depth was calculated by the numbers of stacks.

### Data analysis

Direction and length of narrow structures, manual tracking of the tip of narrow structures, and distribution of amyloplasts in a cell were measured and analysed with the open-source software Fiji (ImageJ 1.54p, Java 1.8.0_322) (Schindelin *et al.*, 2012).

## Results

### Upward elongation of narrow structures in *M. polymorpha* thalli under darkness

**Fig. 1.**
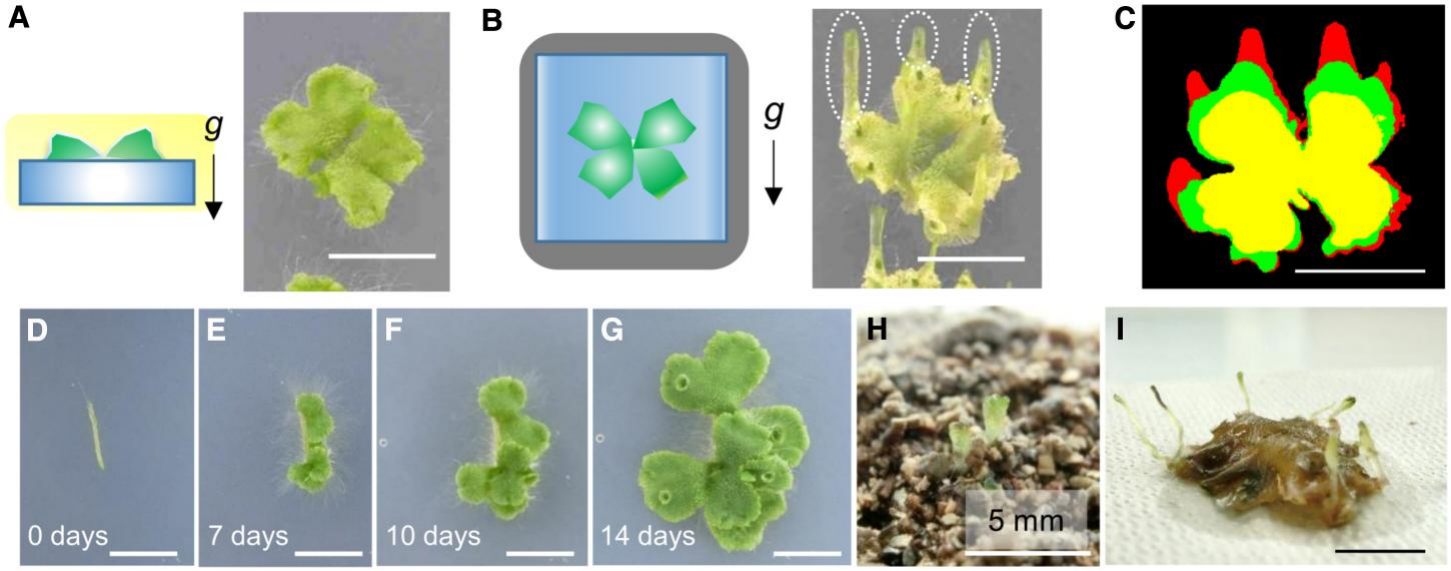
*Marchantia polymorpha* formed upright, narrow structures when grown in the dark. (A, B) Two-week-old wild-type (Tak-1) thalli grown under continuous light (A) were subsequently placed vertically in the dark for an additional 2 weeks (B), with diagrammatic representations provided. The narrow structures are outlined with dotted lines. Black arrows indicate the direction of gravity. (C) Representative morphological changes of thalli after transfer to the dark. The thallus on day 11 is shown at its initial, smallest size (yellow). After transfer to dark conditions, the thallus expands slightly three days later (green), and by seven days, the most pronounced growth, particularly in the apical region (red), is observed. (D–G) A narrow structure dissected from a thallus grown in the dark for 2 months was transferred to the light. Upon exposure, radial tissues developed and thallus-like shapes formed. The number of days after light exposure is shown in the lower left. (H, I) Two-week-old light grown thalli were buried in the soil at a depth of 1 cm. Narrow thalli emerged on the soil surface (H), and the entire plant was excavated 5 weeks after burial (I). Scale bars=1 cm in (A–G, I), 5 mm in (H).

To eliminate the influence of light and to examine the gravity response in thalli of *M. polymorpha*, thalli were grown in the dark. Gemmae of the wild-type plant (Tak-1) were placed on agar plates containing sucrose and grown horizontally under light for 2 weeks (Fig. 1A). Then they were placed vertically in the dark for 2 weeks to examine morphological changes in response to gravity. We found that thin structures (hereafter we call them narrow structures) came out from the thalli and grew in a straight manner upward (Fig. 1B). Then we tracked their growth with time-lapse imaging (Supplementary Movie S1). When 11-day-old thalli grown under light were placed in the dark (Fig. 1C, 0 d; yellow), the tips of the upper parts swelled to form bulges on the third day (Fig. 1C, third day, green). Then, the elongation of the narrow structures accelerated further, and the narrow structures continued to grow upward (Fig. 1C, seventh day, red). In the time-lapse movie, we found that the upper part of the narrow structures appeared to grow faster than the lower part of each thallus (Supplementary Movie S1), presumably because the lower part must bend its body to grow upwards, making its upward growth less straightforward than that of the upper part.

A bulge seemed to form from an apical notch of the thallus at the beginning of the formation process of the narrow structure (Supplementary Fig. S1A). It was elongated in the dark, and the tip of the narrow structure had two apical notches (Supplementary Fig. S1B). Gemma cups were produced on the narrow structures and produced gemmae normally, although the cups were very shallow (Supplementary Fig. S1C, D). The rhizoids on the ventral side and the gemma cups on the narrow thallus’s dorsal side indicate a clear dorsiventral pattern (Supplementary Fig. S1D). Furthermore, when the narrow structure was transferred to light conditions, some parts grew into a shape resembling a thallus (Fig. 1D–G). These results indicate that the narrow structure possesses features of the original thalli.

If the plants were buried in the soil, would they sprout? We buried 2-week-old thalli 1 cm deep in the soil to shade out the light and grew them in the soil (Supplementary Fig. S2). After 5 weeks, we found that some small parts of thalli emerged from the soil (Fig. 1H). When we dug them out, we found that the size of the original thalli had not changed much, but they had lost their green color (Supplementary Fig. S3C, D), whereas newly emerged narrow structures remained green (Fig. 1I, Supplementary Fig. S2D). It was similar to what was observed in the plate experiments in the dark (Fig. 1B). The upward extension of part of the thallus under dark conditions may represent an adaptive trait that enables *M. polymorpha* to survive when buried beneath obstacles.

### Narrow structures respond to gravitational reorientation

**Fig. 2.**
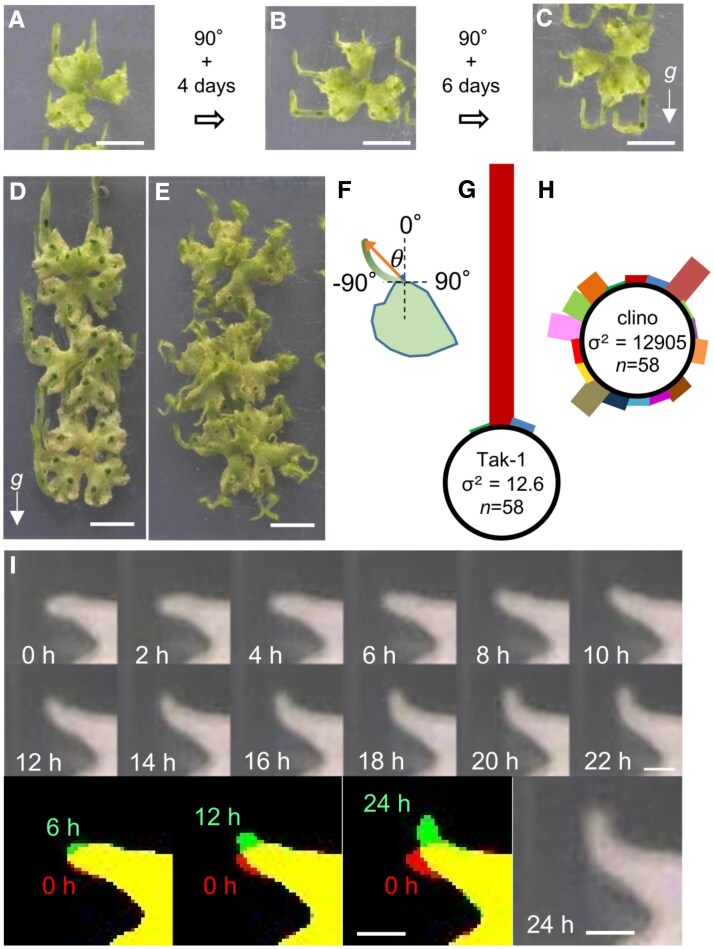
Narrow structures exhibit a sensitive response to gravity. (A–C) The narrow structures displayed negative gravitropism. (A) Seven-day-old gemmalings grown in the light were placed vertically in the dark for 12 d. (B) The thalli with vertically oriented narrow structures were then oriented to 90° and grown for 4 d, resulting in upright elongation. (C) An additional 90° rotation followed by 6 d of growth induced further vertical growth of the narrow structures. (D, E) Thalli grown in the light for 2 weeks were transferred to vertical growth (D) or to a 3D clinostat (E) in the dark for two additional weeks. (F–H) Angles (θ) from the base to the apex of the narrow structures were measured in vertically grown (G) and clinostat-treated (H) Tak-1 plants. The bar length represents the proportion of the narrow structures within each 20° bin relative to the total number. (I) Enlarged images of a tip of the narrow structures. Superimposed images show that when the narrow thallus is rotated 90°, the apex initially faces left. After 6 hours, it exhibits a slight upward curvature, which becomes more pronounced after 12 hours, and by 24 hours the apex is fully oriented upward. Plants were grown for 10 d in the light and then 11 d in the dark in a vertical orientation before the 90° rotation. The elapsed time (h) after rotation is indicated at the bottom of each image. Scale bars=1 cm in (A–E), 2 mm in (I).

We investigated whether the direction of elongation of the narrow structures depends on the direction of gravity. The 7-day-old plants were dark-treated for 12 d to induce the formation of narrow structures (Fig. 2A). When the plants were rotated 90° for 4 d, the narrow structures grew in the direction opposite to that of gravity (Fig. 2B). After another 90° rotation for 4 d, the narrow structures elongated in the direction opposite to gravity again (Fig. 2C).

To further confirm the gravity dependence of the growth direction of narrow structures, we examined that direction when thalli were placed on a 3D clinostat, an experimental device that simulates microgravity environments (Hoson *et al*., 1992). When the thalli were placed vertically in the dark for 2 weeks, they elongated in a straight manner in the direction opposite to that of gravity (Fig. 2D), but when thalli were placed in the 3D clinostat in the dark for 2 weeks, they did not elongate in a straight manner but turned and twisted in various directions (Fig. 2E). We quantified growth angles of narrow structures under gravity on earth (1*×g*) or on the 3D clinostat by measuring the angle formed between the growth direction of the tip and the vertical axis (Fig. 2D–H). We found that most of the narrow structures of the plants placed erect grew in the direction opposite to gravity (Fig. 2D, G); on the contrary, thalli on a 3D clinostat grew irrespective of the directions of gravity (Fig. 2E, H). There was no statistical difference in the growth (length of emerged narrow structures) between treatments (Supplementary Table S4). These results indicate that narrow structures are sensitive to gravity.

To observe morphological changes in the gravity response of narrow structures in detail, the plant with narrow structures grown in the dark for 11 d was rotated 90°, and time-lapse images were taken every 2 h (Fig. 2I; Supplementary Movie S2). All of the narrow structures bent upward after 24 h (Fig. 2I, 0–24 h; Supplementary Movie S2). Detailed observation showed that the tip of the narrow structure changed its direction of growth approximately 6 h after gravistimulation and grew in the direction opposite to gravity after 12 h, and the tip turned straight up after 24 h under our experimental conditions (Fig. 2I; 0–6 h, 0–12 h, and 0–24 h). This result indicates that the response to gravity began to be observed 6 h after gravistimulation, and the direction of elongation was determined until 24 h. After 24 h of gravistimulation, the position of the tip was shifted from the original elongated thallus, suggesting that differential growth of the narrow structure, as well as cell proliferation, causes morphological changes. Therefore, we concluded the gravity response in the elongated thallus of *M. polymorpha* is negative gravitropism.

### Parenchymatous storage cells in narrow structures as the site of amyloplast sedimentation

**Fig. 3.**
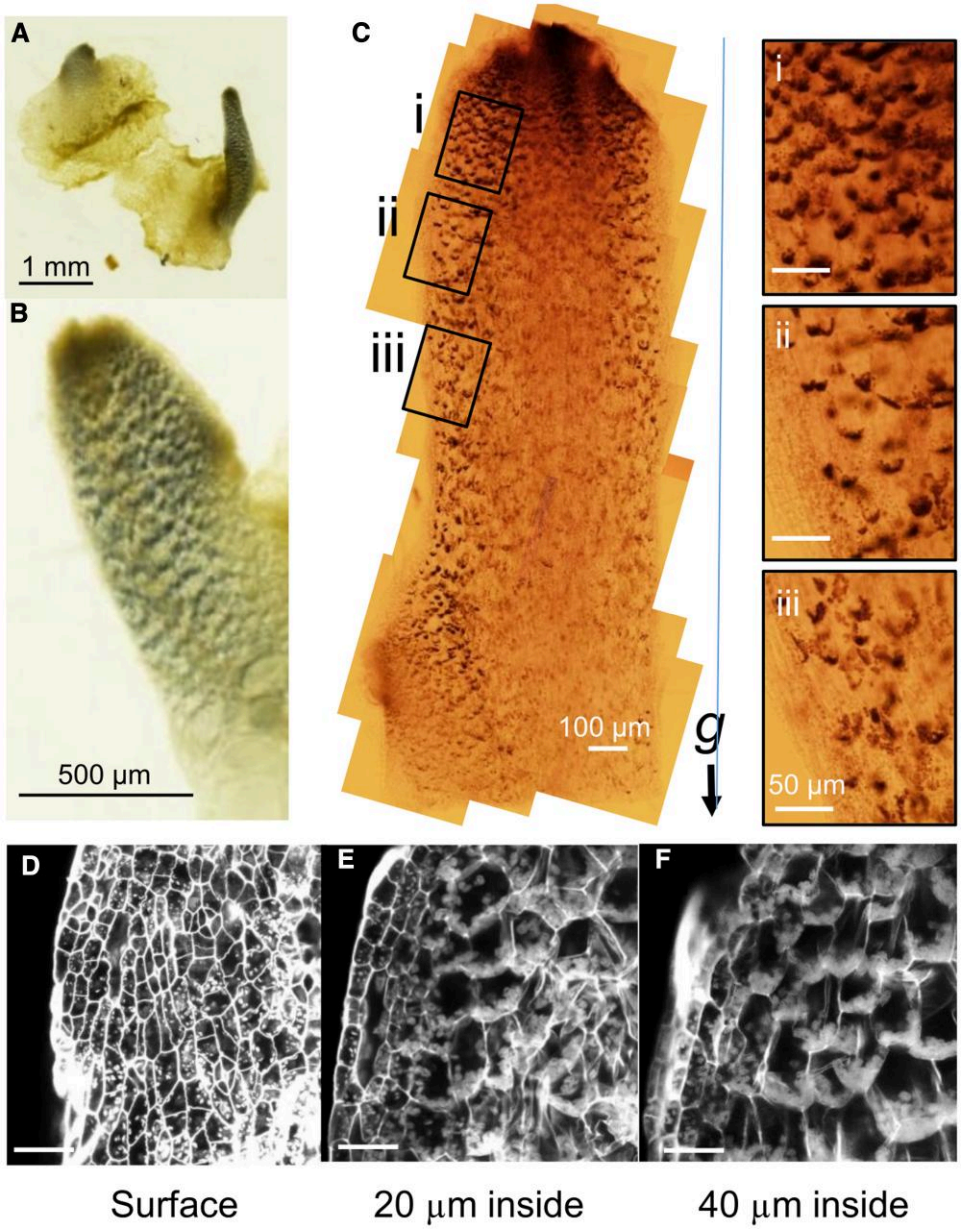
Visualization of starch granules of narrow structures. Samples were stained with Lugol’s solution (A–C) or modified pseudo-Schiff–propidium iodide (D–F). (A, B) Seven-day-old light-grown thalli were transferred to vertical growth in darkness for 14 d. Narrow structures that came out from thalli exhibited dot-like or speckled Lugol staining, whereas the original thalli were not stained. (C) Representative image of Lugol-stained narrow structures from a thallus grown under light for 2 weeks, then vertically in the dark for another 2 weeks. (i–iii) Magnified views of the regions in (C) showing the regions of approximately 200–400 μm (i), 500–700 μm (ii), and 800 μm to 1 mm (iii) from the tip. Starch granules were more abundant and clearly stained near the tip, while signals diminished toward the base. (D–F) Representative images at the surface and 20 and 40 μm below the surface of narrow structures. Small granules in the epidermal cells showed no evident polarity, whereas those in the larger, parenchymatous storage cells beneath them were distributed in a polarized manner. Scale bars=100 μm.

To investigate the involvement of starch granules in the gravitropism of the narrow structure, we performed Lugol’s staining with potassium iodide. Seven-day-old thalli were placed in the dark for 2 weeks, and whole plants were treated with Lugol's solution. Only the narrow structures protruding from the original thallus were stained, suggesting that starch granules were localized exclusively in these structures (Fig. 3A). This indicates that starch granules were present only in the narrow structures but not in the original thallus. The narrow structures extended from the original thalli showed stronger staining at the tip (Fig. 3B). Staining was stronger closer to the tip, with staining becoming sparser and lighter away from the tip (Fig. 3Ci–iii). For each cell, more intense staining was observed on the gravity side, implying that the stained structures are sedimented starch-containing amyloplasts (Fig. 3C).

Modified pseudo-Schiff-propidium iodide staining (mPS-PI) (Haseloff, 2003; Flütsch *et al*., 2018; Zhang *et al*., 2019) was performed to observe the starch-containing amyloplasts in each cell in detail (Fig. 3D–F). On the dorsal epidermis, small intracellular granules were uniformly detected, and these seemed to be undeveloped amyloplasts (Fig. 3D). There was parenchymatous storage tissue under one layer of epidermis (Shimamura, 2016). We found larger amyloplasts settling at the bottom of the cells in the parenchymatous storage tissues (Fig. 3E, F). The cells in the parenchymatous storage tissue lack a layered arrangement and exhibit diverse, irregular shapes in a complex structure. We quantified the polarity of amyloplast distribution in cells located one or two layers beneath the epidermis, since cells in deeper regions showed weak fluorescence, making accurate detection challenging.

### Gravistimulation induces amyloplast sedimentation in cells located at the tips of narrow structures before gravitropic curvature

**Fig. 4.**
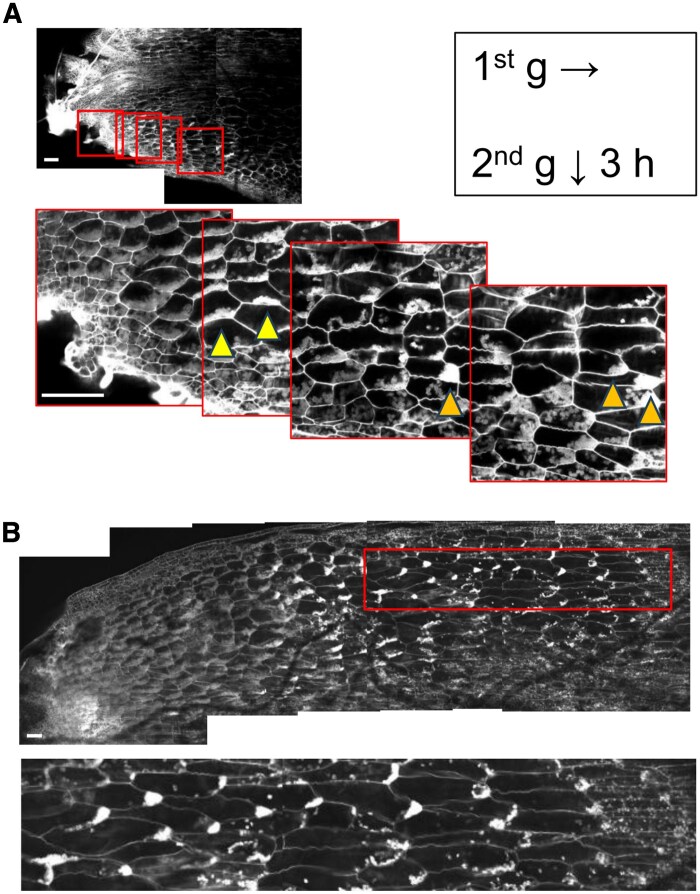
Representative modified pseudo-Schiff–propidium iodide-stained images of amyloplasts in the narrow structure and their magnified views obtained after 3 h of gravitational stimulation. (A) Near the tip, amyloplast sedimentation is observed in the new direction of gravity (two arrwoheads on the apical side), whereas in more distal regions, amyloplasts in some cells remain biased toward the original gravity (three arrowheads at the more basal side). (B) A representative view and magnified view of the region approximately 2 mm from the tip showing that amyloplasts in distal cells remain oriented toward the original gravity direction and become smaller and less abundant further away from the tip. Scale bars=100 μm.

Next, we examined the kinetics of amyloplast relocation in cells of narrow structures upon gravistimulation by 90° reorientation. As bending in response to gravitational stimulation was evident by 6 h, amyloplast sedimentation (Fig. 2), if it plays a role in gravity sensing, should occur within this period. We observed amyloplasts 3 h after the onset of gravitational stimulation. In the apical region of the narrow structure, amyloplasts were tightly packed and showed no apparent polarity. In cells of the subapical region, amyloplasts had sedimented in the new gravity direction, whereas in some cells located farther from the tip, they remained aligned with the original gravity vector (Fig. 4A). In regions more distal from the tip, amyloplasts exhibited a clear polarized distribution, remaining biased toward the original gravity direction (Fig. 4B). To investigate the timing and spatial pattern of amyloplast sedimentation in cells of narrow structures, we analysed its time course changes at subapical regions (200–500 µm from the tip) and distal-subapical regions (between 500 and 1200 µm from the tip).

**Fig. 5.**
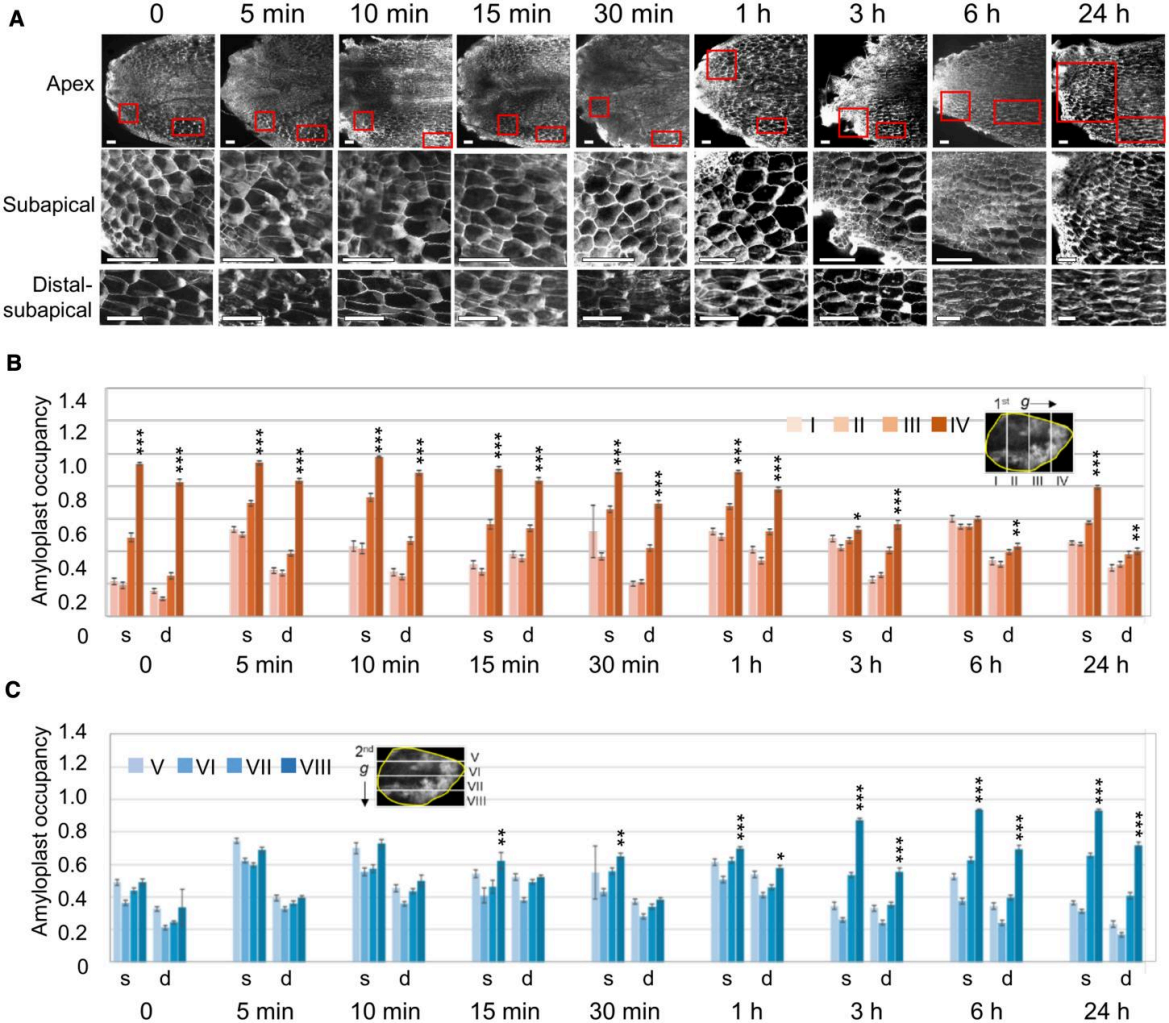
Amyloplast relocation over time in response to gravity stimulation after turning 90°. (A–E) Thalli were grown under 10 d light and 9 d dark vertically (direction of gravity first in B), and then they were rotated 90° (direction of gravity second in C). (A) Representative images of subapical regions (within 500 µm from the tip), and distal-subapical regions (between 500 µm and 1 mm). Scale bar=100 µm. (B, C) Statistical analysis of amyloplast distribution in cells in subapical regions (s) and distal-subapical regions (d). (B) A cell was separated into four regions (I–IV) along the first gravity vector, and the area of amyloplasts in each region was counted and shown as amyloplast occupancy (%). (C) A cell was separated into four regions (V–VIII) along the second gravity vector, and the area of amyloplasts in each region was counted and shown as amyloplast occupancy (%). Each bar indicates the mean ±SE (*n*>100). Statistical significances between I and IV (B) or between V and VIII (C) were evaluated using the Wilcoxon signed-rank test: *P<0.05, **P<0.005, ***P<0.0001.

Quantification of the location of the amyloplast is shown in Fig. 5. The cells were divided into four sections in the direction of gravity to quantify amyloplast distribution. Sections I–IV correspond to divisions based on the original gravity direction, while sections V–VIII correspond to those based on the new gravity direction (insets in Fig. 5B, C). The total area occupied by amyloplasts within each section was measured, and ‘amyloplast occupancy’ was calculated as the proportion of area occupied by amyloplasts in each section (Fig. 5). To evaluate whether amyloplasts had sedimented to the bottom of the cells, we analysed statistical differences between layers I and IV, and between layers V and VIII.

Before the second gravitational stimulation, a clear polarization of amyloplasts along the initial gravity direction was observed even in cells very close to the tip (Fig. 5A; time 0). This was also supported by the large difference in amyloplast occupancy between region I and region VI, while no significant difference was found between region V and region VIII (Fig. 5B, C; time 0). After 5 min of gravistimulation, amyloplast occupancy in regions II, III, VI, and VII at the subapical region of the cells increased, indicating amyloplasts started to disperse at the subapical region (Fig. 5A; 5 min). No clear differences were observed between the 5 min and 10 min time points, but accumulation of amyloplasts in the new gravity direction began to appear at the subapical region between 15 and 30 min after gravistimulation (Fig. 5C). By 1 h, amyloplast polarization toward the new gravity direction had also begun to appear in the distal apical region (Fig. 5C). In the subapical region, sedimentation of amyloplasts in the initial gravity direction was partially relieved at 3 h, and by 6 h no such sedimentation was observed (Fig. 5B). In parallel, amyloplast sedimentation toward the new gravity vector became pronounced in the subapical region by 3 h and continued to progress over time in both the subapical and distal subapical regions (Fig. 5C). At 24 h, visual inspection confirmed amyloplast sedimentation in the new gravity direction in both the subapical and distal subapical regions (Fig. 5A, C). However, in the subapical region amyloplasts appeared to have accumulated along the original gravity direction (Fig. 5B). This is likely due to cell elongation occurring in region I in an oblique upward direction during this period, resulting in the amyloplasts that had sedimented in the new gravity direction now appearing predominantly in region IV. In summary, amyloplast sedimentation in the narrow thallus occurs in parenchyma cells located relatively close to the apex. After gravistimulation, amyloplasts begin to shift within 5 min, but clear sedimentation along the new gravity vector becomes evident around 15 min. This process initiates earlier in cells near the apex and takes longer in more distal cells. By 3 h, complete sedimentation is observed near the tip, preceding the onset of growth reorientation at 6 h. In more distal regions, sedimentation in the new gravity direction is less pronounced, and amyloplasts are smaller and fewer in number. These findings suggest that gravity sensing primarily occurs in the subapical region.

### Generation of starchless mutants in *M. polymorpha*

**Fig. 6.**
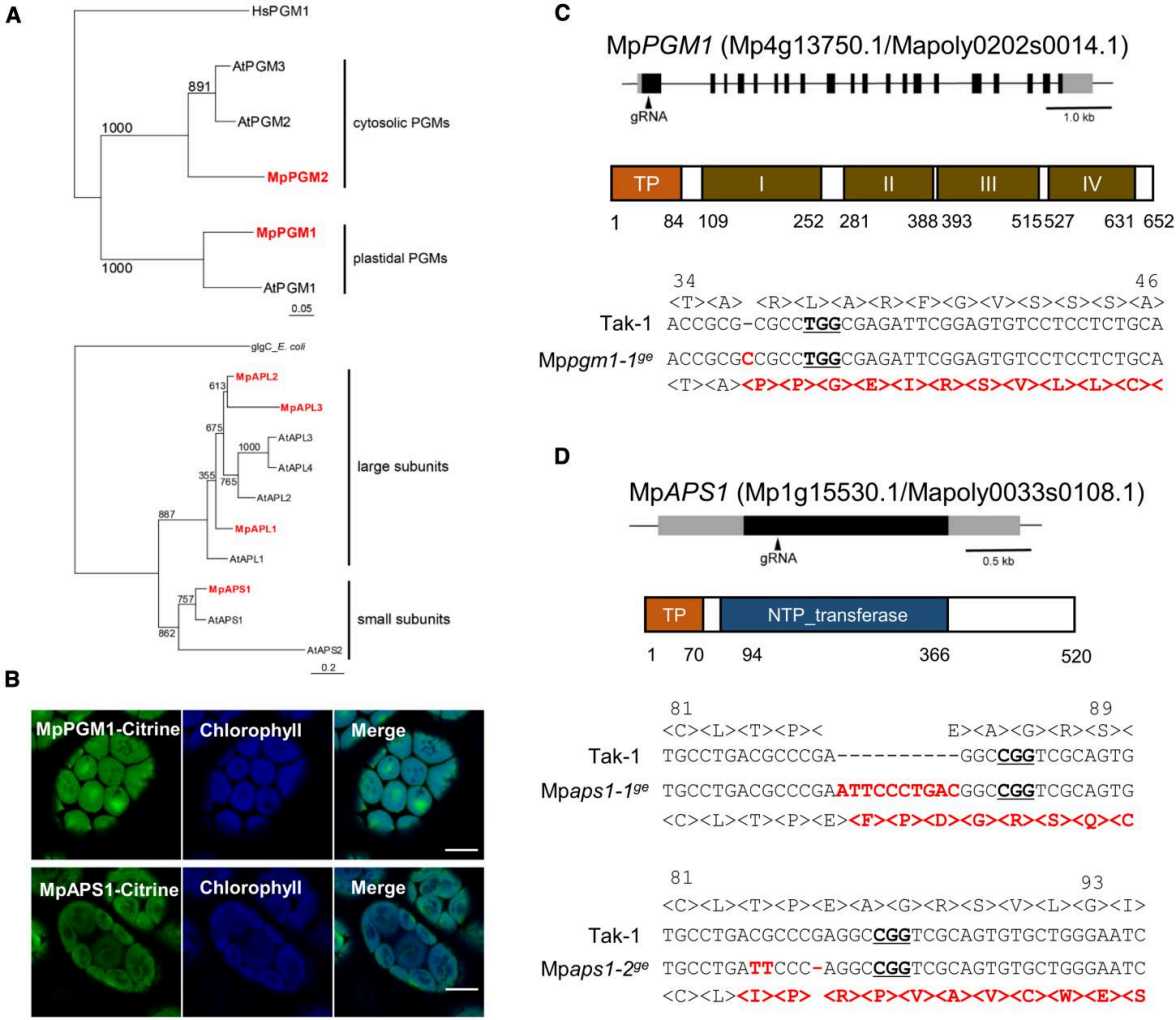
Starchless mutants in *M. polymorpha* Tak-1 produced by CRISPR-Cas9 method. (A) The maximum-likelihood phylogenetic tree of phosphoglucomutase (PGM) and small/large subunits of ADP-glucose pyrophosphorylase (APS/APL). The maximum-likelihood phylogenetic analysis was performed using sequences of PGM, APS, and APL in Arabidopsis and *M. polymorpha*. As an outgroup to these, *Homo sapiens* PGM (for PGM) or *Escherichia coli* glgC (for APS/APL) was used. Bootstrap=1000. (B) Localization of MpPGM1 (top) and MpAPS1 (bottom). Green and blue pseudo-colors indicated fluorescence from Citrine and chlorophyll, respectively. Scale bars=10 μm. (C) Mp*PGM1* gene structure and mutation site in the *Mppgm1-1* mutant. Top, gray and black boxes indicate untranslated and coding regions, respectively, and arrowheads indicate the region of the used guide RNA (gRNA). Middle, domain sequences of MpPGM1. MpPGM1 possesses the transit peptide at the N-terminus and the PGM_PMM_I (PF02878), PGM_PMM_II (PF02879), PGM_PMM_III (PF02880), and PGM_PMM_IV (PF00408) domains. Bottom, the genome and translated amino acid sequences of Mp*PGM1* genes in Tak-1 (wild type) and Mp*pgm1-1^ge^*. The PAM sequence for gRNA is underlined, and red letters indicated the mutation sites in Mp*pgm1-1^ge^*. (D) Mp*APS1* gene structure and mutation sites in the Mp*aps1-1* and Mp*aps1-2* mutants. Top, gray and black boxes indicate untranslated and coding regions, respectively, and arrowheads indicate the region of gRNA. Middle, domain sequences of Mp*APS1*. Mp*APS1* possesses the transit peptide at the N-terminus and the NTP_transferase (PF00483) domain. Bottom, the genome and translated amino acid sequences of MpAPS1 genes in Tak-1, Mp*aps1-1^ge^* and Mp*aps1-2^ge^*. The PAM sequence for gRNA is underlined, and red letters indicated the mutation sites in Mp*aps1-1^ge^* and Mp*aps1-2^ge^*.

To verify whether the settling of amyloplasts is necessary for sensing gravity in *M. polymorpha*, we generated mutants lacking amyloplasts. Representative starchless mutants *pgm* and *aps1* in Arabidopsis, which are deficient in plastid phosphoglucomutase and a small subunit of ADP-glucose pyrophosphorylase, required for the full development of amyloplasts, have reduced gravitropic response in both roots and shoots (Caspar and Pickard, 1989; Kiss *et al.*, 1989,1996; Weise and Kiss, 1999; Vitha *et al.*, 2000). Therefore, we first identified homologs of these genes in *M. polymorpha*. Arabidopsis has three *PGM* genes (*AtPGM1–3*). Only AtPGM1 is localized in the plastid and required for starch biosynthesis (Caspar *et al*., 1985). In the *M. polymorpha* genome, we identified two homologs of *PGM* genes (Fig. 6A). The phylogenetic analysis suggested that MpPGM1 is a counterpart of AtPGM1, and MpPGM2 is a counterpart of AtPGM2/3 (Fig. 6A). Indeed, MpPGM1 but not MpPGM2 possesses the transit peptide, and Citrine-tagged MpPGM1 (MpPGM1–Citrine) was localized in the plastid, as in our previous observation (Norizuki *et al*., 2023; Fig. 6B, C). Therefore, MpPGM1 could be involved in starch biosynthesis in plastids. ADP-glucose pyrophosphorylase is a heterotetramer (two large subunits and two small subunits) and Arabidopsis possesses two small subunit genes (At*APS1*, At*APS2*) and four large subunit genes (At*APL1–4*) (Villand *et al*., 1993). In the *M. polymorpha* genome, one small subunit gene (Mp*APS1*) and three large subunit genes (Mp*APL1–3*) were detected (Fig. 6A). MpAPS1 possesses the transit peptide and Citrine-tagged MpAPS1 was localized in the plastid (Fig. 6B, D). Taken together, we chose Mp*PGM1* and Mp*APS1* genes to generate starchless *M. polymorpha* mutant lines.

**Fig. 7.**
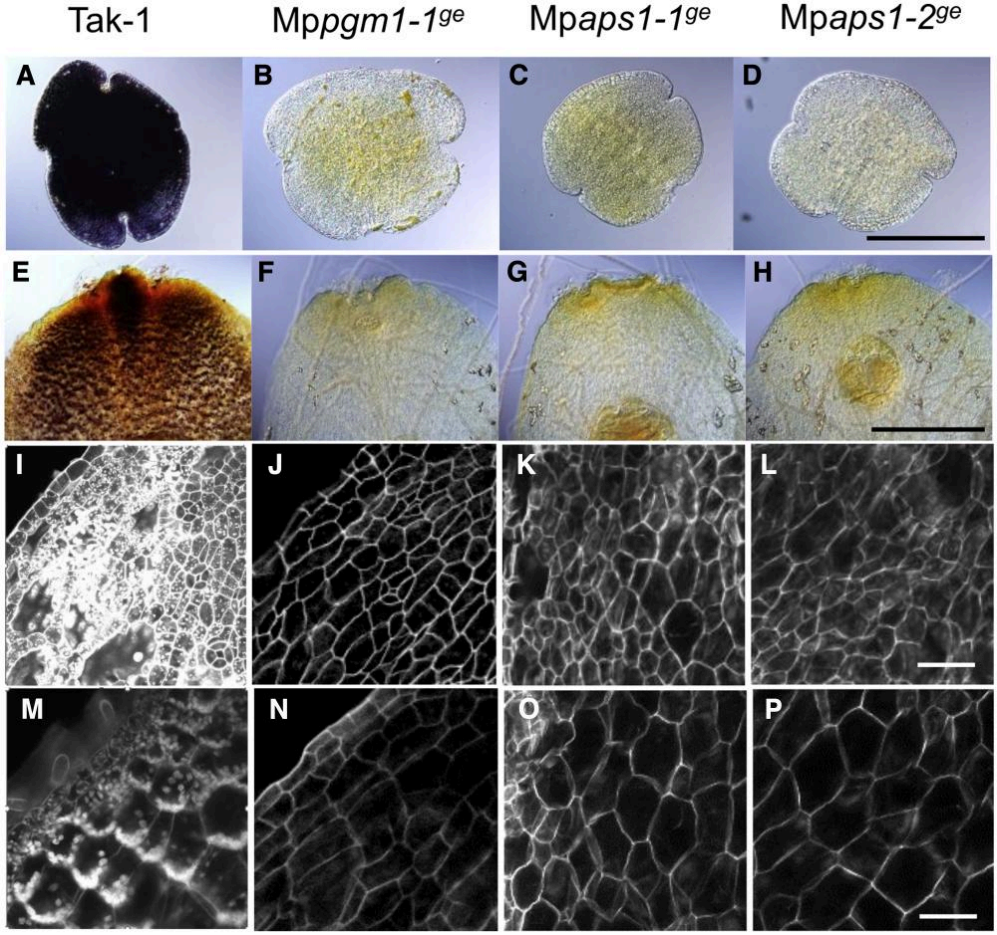
Starch stain in the wild-type plant, Mp*pgm1-1^ge^*, Mp*aps1-1^ge^*, and Mp*aps1-2^ge^*mutants. (A–H) Starch of gemmae (A–D) and narrow structures (E–H) was visualized using Lugol’s solution. Narrow structures were obtained from thalli grown under light for 11 d and then vertically in the dark for 6 d. (I–P) Narrow structures stained with modified pseudo-Schiff–propidium iodide. Optical sections of surface (I–L) and 40 µm beneath the surface (M–P) of the narrow structures. Scale bars=200 µm in (A–D), 100 µm in (E–H), 50 µm in (I–P).

Using the CRISPR/Cas9 system (Sugano *et al*., 2018), we obtained one Mp*pgm1* and two Mp*aps1* mutant lines with frame-shift mutations (Mp*pgm1-1*^ge^, Mp*aps1-1*^ge^, and Mp*aps1-2*^ge^; Fig. 6C, D). We first examined when and where Lugol’s staining was detected in wild-type plants. In the wild type, the whole plant body was strongly stained in gemmalings on days 0 and 1, but became speckled on days 2 and 3 (Supplementary Fig. S3). The Lugol stain was limited to the center and notch areas after day 4 (Supplementary Fig. S3). We checked Lugol’s staining in gemmae and narrow structures from the wild type Tak-1, Mp*pgm1-1^ge^*, Mp*aps1-1^ge^*, and Mp*aps1-2^ge^*. There was blue–purple coloring in the wild type Tak-1, but not in Mp*pgm1-1^ge^*, Mp*aps1-1^ge^*, and Mp*aps1-2^ge^* (Fig. 7A–H). Further detailed examination by mPS-PI staining showed that the signals were observed both on the surface and at a depth of 40 µm of the wild-type narrow structures (Fig. 7I, M), whereas starch grains were not detected in Mp*pgm1* and Mp*aps1* mutants (Fig. 7J–L, N–P), showing that these mutants are starchless and do not have sedimentable amyloplasts.

### Starch-containing amyloplasts were required for full gravitropism in *M. polymorpha*

**Fig. 8.**
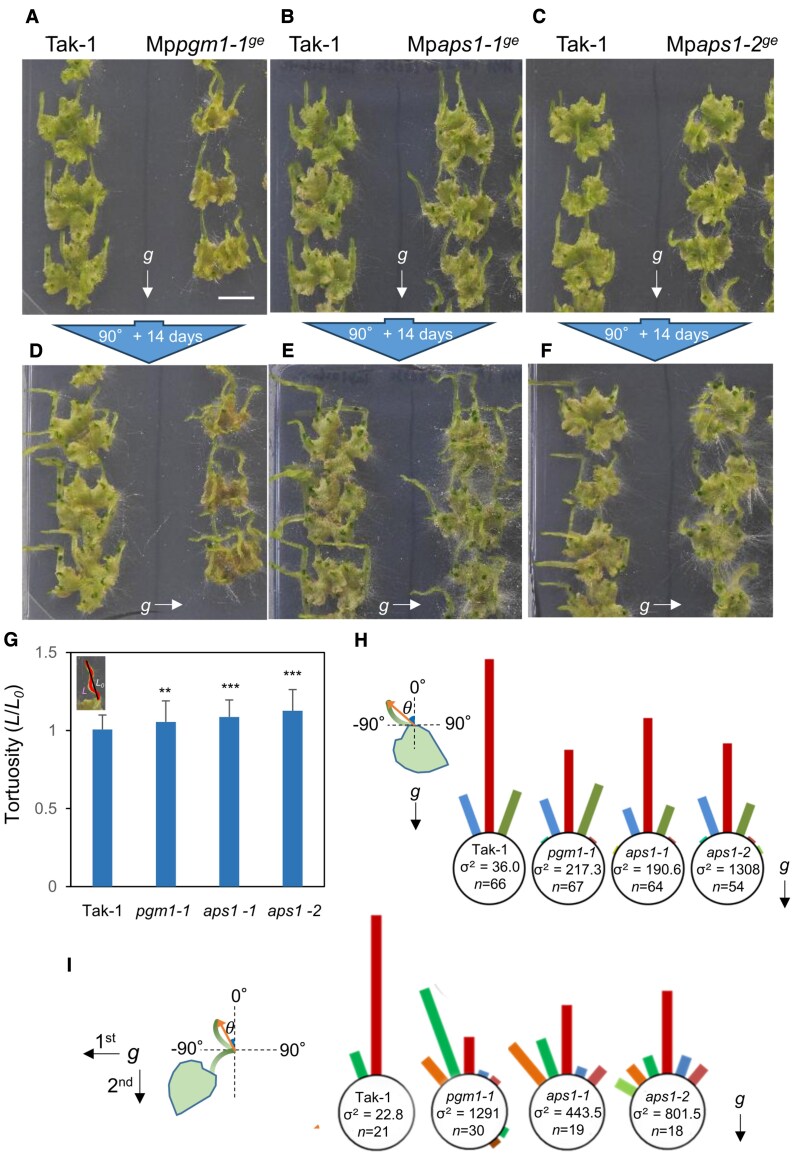
Narrow structures of starchless mutants were wavy, with greater variation in the elongation direction. (A–C) Wild type and Mp*pgm1-1^ge^* (A), Mp*aps1-1^ge^* (B), and Mp*aps1-2^ge^* (C) were grown under light for 2 weeks and grown vertically in the dark for another 2 weeks. (D–F) They were subsequently rotated 90° and grown vertically for an additional 14 d in darkness. The leftmost lanes of each plate contain wild-type Tak-1 plants as controls. (G) Tortuosity of narrow structures in wild type and starchless mutants. Tortuosity was calculated as by the ratio of the actual length shown in a curved line(*L*) to the straight distance from the basal position of the narrow structures to the tip (*L*_0_). Kruskal–Wallis rank sum test followed by Steel–Dwass: **P<0.01, ***P<0.001, *n*>54, mean ±SD. (H) Measurements of the angle (θ) from the basal position to the tip of the narrow structure. The bar length represents the proportion of the narrow structures within each 20° bin relative to the total number. (I) Measurement of the angle (θ) between the tip position before gravistimulation and the tip position 14 d after 90° rotation. Scale bar=1 cm.

Wild type, Mp*pgm1*, and Mp*aps1* were grown for 2 weeks in light and grown vertically for 2 weeks in the dark. Narrow structures were formed, but unlike the wild-type plant, those of each mutant were not straight but helical and bent (Fig. 8A–C). To quantify the degrees of bending, the tortuosity, the length of the narrow structures (*L*)/the shortest distance from the base to the top of the narrow structures (*L*_0_), was evaluated. The value of tortuosity was almost 1 in the wild type indicating straight growth, but was larger in Mp*pgm1-1^ge^*, Mp*aps1-1^ge^*, and Mp*aps1-2^ge^* (Fig. 8G). The length of narrow structures in the wild-type plants, Mp*pgm1-1^ge^*, Mp*aps1-1^ge^*, and Mp*aps1-2^ge^* were not significantly different (Supplementary Table S5), indicating that the narrow structures of the starchless mutants tended to be frizzier than those of the wild type. The angles from the base to the top of the narrow structures were mostly opposed to the direction of gravity in the wild-type plants (Fig. 8H). The starchless mutants also had the highest frequency of growth in the direction opposite to that of gravity, but larger σ values represent greater variation in the growth angle (Fig. 8H).

The gravitropic responses of narrow structures of starchless mutants were further examined when plants were rotated by 90° (Fig. 8D–F). The narrow structures of the wild type elongated in the direction opposite to that of gravity ranging from −15.8° to 2.1°, whereas the starchless mutants elongated in various directions ranging from −44.0 to 143.7°, −43.0 to 27.0° and −54.5 to 45° in Mp*pgm1-1^ge^*, Mp*aps1-1^ge^*, and Mp*aps1-2^ge^*, respectively (Fig. 8I). A much higher degree of variability in the growth direction of narrow structures was observed in the starchless mutants after reorientation.

**Fig. 9.**
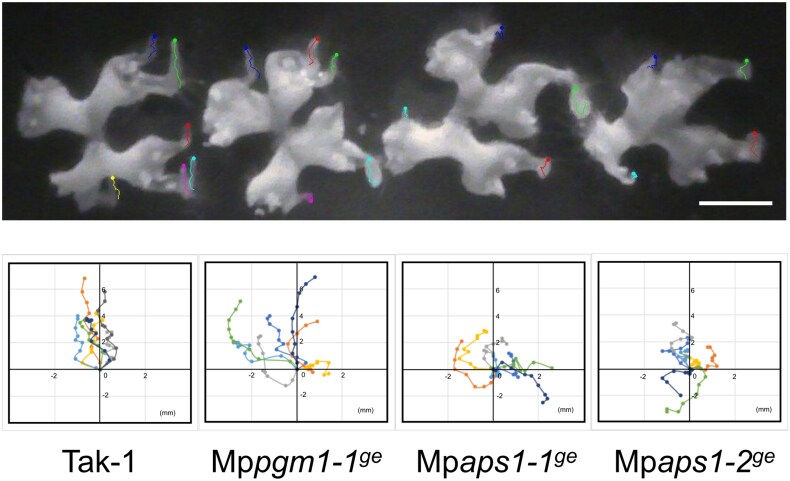
Tip movement traces of narrow structures in wild-type plant and starchless mutants (Mp*pgm1-1^ge^*, Mp*aps1-1^ge^*, and Mp*aps1-2^ge^*) following 90° gravistimulation. Thalli were grown under light for 14 d and then vertically in the dark for 12 d before being rotated by 90°. Tip positions were recorded every 12 h over a period of 4.5 d. *n*=7. Scale bar=1 cm. Graphs show two-dimensional movement trajectories, with the initial position set as the origin [(*x*, *y*)=(0, 0)].

The reduced gravitropic phenotype of the starchless mutants may be most pronounced when the direction of gravity was changed, so we captured the dynamics of the starchless mutant when subjected to a 90° gravitational stimulus (Fig. 9; Supplementary Movie S3). The movements of the apical top of the narrow structures were quantitatively analysed by single-particle tracking (Fig. 9). In the case of wild-type plants, the narrow structures elongated to the top with very slight side-to-side movements, but the swing was mostly within 1 mm to the left or right. On the other hand, starchless mutants moved more widely, and moreover, many of them moved in large arcs and/or sometimes grew in the direction of gravity showing altered gravitropism (Fig. 9; Supplementary Movie S3). The absence of starch likely impairs the ability to take the shortest upward path, suggesting that it takes more time to recognize the correct upward direction. These results demonstrated that starch-containing amyloplasts were required for full gravitropism of thallus in *M. polymorpha* as with vascular plants such as Arabidopsis.

## Discussion

### Narrow structure is an ideal material for studying gravitropism in *M. polymorpha*

We have found that structures extended from the thalli of *M. polymorpha* grew upward when they were subjected to dark conditions (Fig. 1). This direction of elongation was always opposite to gravity in response to changes in gravity directions, but 3D clinostat treatment disrupted the gravity response, resulting in the narrow structures wiggling and curving in various directions (Fig. 2). These results demonstrated that the narrow structures were highly sensitive to gravity. In previous studies, gametangiophore stalks have been used as material for gravitropic responses in *M. polymorpha* (Kato *et al*., 2015; Fisher *et al*., 2023). Gametangiophore stalks, like the narrow thallus, contain amyloplast-rich tissues and bend upward when placed horizontally. However, unlike the narrow thallus, gametangiophore stalks are thicker and sometimes twist during growth, which prevents them from consistently growing straight. This may be one of the contributing factors to the variability in both the degree and the position of curvature in response to gravity—sometimes occurring directly beneath the archegoniophore, and at other times at a slightly more distal location—resulting in inconsistent bending patterns. Rhizoids have also been suggested as potential sites of gravitropic response in *M. polymorpha* (Fisher *et al*., 2023). However, in our experiments, rhizoids were not stained with iodine solution (Supplementary Fig. S3), suggesting that they do not contain starch granules. Furthermore, while the narrow structure consistently grows upright in the direction opposite to gravity, the rhizoids emerging from it tend to grow perpendicular to it (Supplementary Fig. S1D). This growth is not oriented in the direction of gravity, nor do the rhizoids bend in response to changes in gravitational direction. This indicates that the growth direction of rhizoids may depend on the specific position on the thallus from which they emerge, as well as the orientation of the thallus itself.

The narrow structure, with its ability to repeatedly change its growth direction in response to gravity (Fig. 2A–C), appears to be the most sensitive structure for studying gravitropism in *M. polymorpha*. Its formation and gravitropic response are highly reproducible, allowing simultaneous observation and comparison of multiple individuals, as demonstrated in time-lapse videos (Supplementary Movies S1–S3). Moreover, while gametangiophore stalk formation requires far-red light irradiation system, the narrow thallus can be induced in darkness, making it a more accessible and practical material for gravitropic studies.

### Dark-induced apical growth potentially involving auxin signaling

When thalli were placed in an environment without light, the original thalli stopped growing and faded in color in the dark, whereas the narrow structures were green and actively elongating (Fig. 1H, I; Supplementary Fig. S2), which may be due to the supply of energy and nutrients from the original thalli that accompanies the formation of the actively dividing parts (narrow structures). The narrow structures have almost the same properties as the general thalli except for a narrow body with one-directional elongation, and shallow or barely visible rims of the gemma cups on the narrow structures (Supplementary Fig. S1). Auxin is one of the factors inducing the elongation of rims of gemma cups (Kato *et al*., 2015), so the shallow gemma cups on narrow thallus may result from the lower level of auxin or lower sensitivity to it. Excision of the apex fully provoked regeneration and a transient decrease in endogenous auxin is involved in initiating cell cycle re-entry during regeneration (Ishida *et al*., 2022). Darkness may have a similar effect to regeneration after excitation, causing a reduction in auxin levels and thus increasing growth activity at the apical tip.

### Presence of amyloplasts in specific tissues and their sedimentation in gravity direction

It has been reported that *M. polymorpha* thalli collected from the field and treated with one day of darkness showed unilateral distribution of Lugol’s staining within the cells of the ground tissue, including both the thalli and gametophytes (Němec̀, 1900; Petschow, 1933). We also detected sedimented amyloplasts in narrow structures by Lugol’s staining, but no signal was found in the original thalli placed in the dark for 2 weeks (Fig. 2A). We also showed that gemmae obtained from gemma cups were stained well overall, but the color faded after 2 d, and nothing but apical notches were stained after 4 d (Supplementary Fig. S3). These results indicate that starch granules are not ubiquitously present, but rather appear transiently at specific developmental stages and localized regions. Notably, the strongly stained tissues correspond to key sites involved in gravity perception, such as those responsible for establishing dorsoventral polarity (e.g. gemmae) and determining growth orientation (e.g. apical notches) (Fitting, 1936; Halbsguth, 1953). This localization pattern is consistent with the presence of amyloplasts functioning as statoliths. The Lugol’s staining in narrow structures appeared to be biased toward the bottom of the cell; this pattern was very similar to that reported in the statocyte endodermal cells of Arabidopsis flower stalks, also suggesting a function of starch granules as potential statoliths (Fig. 3; Weise and Kiss, 1999). The mPS-PI method also detected amyloplasts settled in the direction of the gravity vector in parenchymatous storage cells of the narrow structures (Fig. 3E, F), suggesting that those cells are candidate statocytes. Unlike endodermal cells, these parenchymatous cells are not arranged in a single layer but instead form multiple layers that extend throughout most of the narrow structure, excluding the epidermis, rhizoids, and the region near the midrib (Shimamura, 2016). In the apical portion of the narrow structure, well-developed amyloplasts and their sedimentation are clearly observed. We examined the intracellular position of amyloplasts in the narrow structures 3 h after gravistimulation by 90° reorientation. Importantly, amyloplasts did not sediment uniformly across all cells; rather, the rate of sedimentation in the direction of the new gravity vector gradually decreased with increasing distance from the apex, and amyloplasts tended to remain in their original sedimentation positions (Fig. 4A). In more distal regions, both the number and the size of amyloplasts amyloplasts gradually decrease, and sedimentation becomes undetectable (Fig. 4B), consistent with the results of the iodine–starch reaction (Figs. 3C). These findings highlight several distinct characteristics of *M. polymorpha* compared with seed plants. Parenchymatous storage cells closer to the apex appear to function as statocytes, in which well-developed amyloplasts sediment in response to gravity. In contrast, as the distance from the apex increases, starch accumulation decreases, potentially reducing the density of amyloplasts and thereby preventing their sedimentation in the direction of gravity.

### Temporal changes in amyloplast positioning in response to gravistimulation

To investigate the dynamics of amyloplasts in response to gravity, in parenchymatous storage cells, we applied a gravitropic stimulus by rotating the sample 90° and analysed the time course of amyloplast positions (Fig. 5). In the narrow structure, amyloplast sedimentation begins within 5 min after gravistimulation suggesting that the relocation of amyloplasts begins within this early time window (Fig. 5C). However, the accumulation of amyloplasts at the bottom of the cell becomes apparent only after approximately 15–30 min following stimulation. Since the amyloplast occupancy in regions VI and VII remains high until 1 h after stimulation, it is likely that a substantial proportion of amyloplasts have not yet fully sedimented to the bottom at this stage (Fig. 5C). By 3 h, the occupancy in regions V, VI, and VII markedly decreases, while that in region VIII increases, indicating that most amyloplasts have sedimented to the new bottom of the cells (Fig. 5C). At 6 h, when curvature of the thallus begins (Fig. 2), sedimentation toward the original gravity direction is no longer observed in the subapical region (Fig. 5B), and amyloplasts are clearly accumulated along the new gravity vector (Fig. 5C). At 24 h after gravistimulation, amyloplast sedimentation was observed in most cells within both the subapical and distal-subapical regions (Fig. 5A). The fact that amyloplast sedimentation preceded gravitropic bending is consistent with the starch-statolith hypothesis. It has been reported that sedimentation of amyloplasts in shoot endodermal cells of Arabidopsis was observed within 3 min at 90° reorientation (Saito *et al*., 2005), and Arabidopsis flowering stalks achieved 60° curvature in 80 min (Caspar and Pickard, 1989). In contrast, the sedimentation of amyloplasts (started at 15 min and completed at 3 h) and response (started at 6 h) observed in the narrow structures of *M. polymorpha* appear to be considerably slower (Figs 2, 5). One possible reason for the delayed gravitropic response in *M. polymorpha* may be the slow sedimentation of amyloplasts. This may indicate that the amyloplasts in *M. polymorpha* cells have a relatively low density, or that there may be physical constraints within the cell that impede their sedimentation.

In roots or rhizoids, gravitropic bending in seed plants after a 90° reorientation of the seedlings is faster than that of basal vascular plants (ferns and lycophytes), and non-vascular plants (mosses) (Zhang *et al*., 2019). Furthermore, it has been reported that cells containing amyloplasts are distributed more broadly in vascular and non-vascular plants compared with seed plants (Zhang *et al*., 2019). Similar to the root system, it is possible that, during evolution, amyloplast development in shoots or thalli also became confined to specific cells, which in turn evolved intracellular environments that facilitated more efficient sedimentation.

### Conserved role of amyloplasts as statoliths in *M. polymorpha* and seed plants

Gravity-sensing mechanisms vary across different organisms. In the unicellular green alga *Chlamydomonas reinhardtii*, gravity is sensed by the entire cell body rather than by the starch-covered pyrenoid (Roberts, 2006). In *Chara globularis*, gravity perception is mediated by barium sulfate vesicles instead of starch-filled amyloplasts (Limbach *et al*., 2005). In contrast, the protonemata or caulonemata of mosses possess sedimentable starch-filled amyloplasts at their growing tips, which have been suggested to function as statoliths (Jenkins *et al.*, 1986; Walker and Sack, 1990; Schwuchow *et al*., 1995; Sack *et al*., 2001). Many terrestrial plants examined so far use starch grains (amyloplasts) for gravitropism, suggesting their common role as statoliths for gravity perception in land plants (Barlow, 1995; Sievers *et al.*, 1996; Sack *et al.*, 2001; Yoder *et al.*, 2001). Sufficient evidence for the role of the amyloplasts as statoliths has been obtained by using intracellular magnetophoresis to move them laterally in various plant organs and induce a gravitropic response (Kuznetsov and Hasenstein, 1997; Weise *et al*., 2000). To examine whether amyloplast sedimentation contributes to gravity sensing in *M. polymorpha*, we generated starch-deficient Mp*pgm1* and Mp*aps1* mutants with no starch signals (Figs 6, 7). Unlike the straight vertical growing narrow structures of the wild-type plants, those of the starchless mutants exhibited curved or spiral upward growth with greater variability and significantly higher tortuosity (Fig. 8A–C, G, H). These results are consistent with those reported for starchless mutants of Arabidopsis (*pgm1* and *aps1*), in which vertical growth of both shoot and root is impaired, and directional variation is increased compared with the wild type (Vitha *et al*., 2000). The alteration of growth direction in the narrow structures of starchless mutants became particularly evident within the first 24 h following gravistimulation (Fig. 9; Supplementary Movie S3). These observations emphasize that sedimentation of amyloplasts is essential for proper gravitropic responses in *M. polymorpha*. However, starchless mutants including Mp*pgm1* and Mp*aps1* can respond to gravity to some extent, and they eventually move upward after a sufficient time (approximately over 24 h) after gravistimulation, suggesting that there may be an amyloplast-independent pathway for the gravitropism in *M. polymorpha* (Fig. 8). This observation is different from that of clinostat-treated plants, which completely lack polarity in their growth direction (Fig. 2E). Previous studies using starchless mutants of Arabidopsis have shown that gravitropism is not entirely abolished even in the absence of starch (Caspar and Pickard, 1989; Kiss *et al.*, 1989, 1996; Kiss, 2000; Morita, 2010). Plastids not accumulating starch granules might be a reasonable candidate for statoliths albeit the function is very weak, assuming that rapid and extensive plastid sedimentation is not necessary for gravity sensing. A candidate of the non-statolith system is the mass of the entire protoplast. This ‘protoplast pressure hypothesis’ is a mechanism that senses pressure due to the weight of the cytoplasm acting on the membrane and cell wall, and is thought to be an early evolved and probably less sensitive one (Wayne *et al*., 1990; Weise and Kiss, 1999). Multiple pathways may co-exist in gravitropism in land plants (Barlow, 1995).

Further studies are needed to elucidate the mechanisms of gravitropism in each plant, why the sedimentation speed of amyloplasts differs among species, and how the intracellular structure evolves to facilitate gravity sensing. Comparative gravitropic studies using a wide range of experimental model plants would help to elucidate the basic mechanism acquired by the ancestral terrestrial plants and their diversification during land plant evolution.

## Supplementary Material

eraf375_Supplementary_Data

## Data Availability

The primary data supporting this study were not made publicly available at the time of publication. The data that support the findings of this study are available from the corresponding author upon request.

## Supplementary data

The following supplementary data are available at *JXB*  online.

Fig. S1. Narrow structures retained some traits as thallus.

Fig. S2. Narrow structures emerged from thalli buried in the soil.

Fig. S3. Young gemmalings lose starch accumulation as they develop.

Table S1. The sequences used in the phylogenetic analysis of PGM

Table S2. The sequences used in the phylogenetic analysis of APL

Table S3. Primers used in this study

Table S4. The length of narrow structures treated with and without clinorotation

Table S5. The length of narrow structures in wild type and starchless mutants

Movie S1. Time-lapse images of 11-day-old light-grown thalli transferred to the dark.

Movie S2. Time-lapse images of narrow structures growing after 90° rotation in darkness.

Movie S3. Time-lapse movie of narrow structures of wild type, Mp*pgm1-1^ge^*, Mp*aps1-1^ge^*, and Mp*aps1-2^ge^* with tip traces as shown in Fig. 9.

## Abbreviations

APL: large subunit of ADP-glucose pyrophosphorylase
APS: small subunit of ADP-glucose pyrophosphorylase
mPS-PI: modified pseudo-Schiff–propidium iodide
PGM: phosphoglucomutase
