# Supplementary material for: Amyloplasts are necessary for full gravitropism in thallus of Marchantia polymorpha
Source: J Exp Bot. 2025 Aug 19;76(22):6741–57. doi: 10.1093/jxb/eraf375 (PMC12675261; doi:10.1093/jxb/eraf375)
Supplement: eraf375_Supplementary_Data [file eraf375_supplementary_data.zip › jexbot314494-file001.pdf]

Supplementary Data

Amyloplasts are necessary for full gravitropic sensitivity in *M. polymorpha*

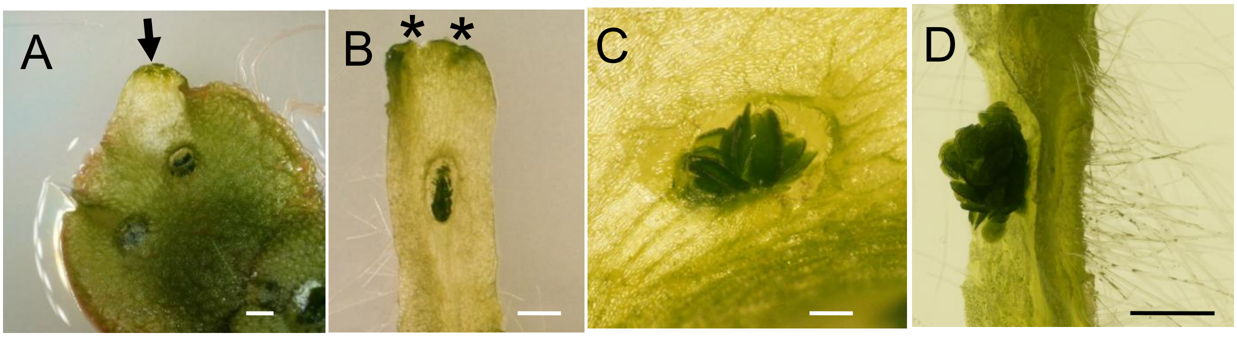

Fig. S1

Narrow structures retained some traits as thallus. (A) A bud structure (black arrow) emerged from the apical notch of a thallus grown for 2 weeks in the light and 5 days in the dark. (B) Narrow structures grew straight, typically possessing one or two apical notches (asterisks) at the apex. (C, D) Developed narrow structures produced some gemma cups along the midrib. Developing (C) and mature (D) gemma cups on the narrow structures with very shallow rim. (D) The narrow structures exhibited clear dorsiventrality; Gemma cups formed on the dorsal side, while rhizoids covered the ventral surface. Scale bars = 1 mm in A, B, and D; 200 μm in C.

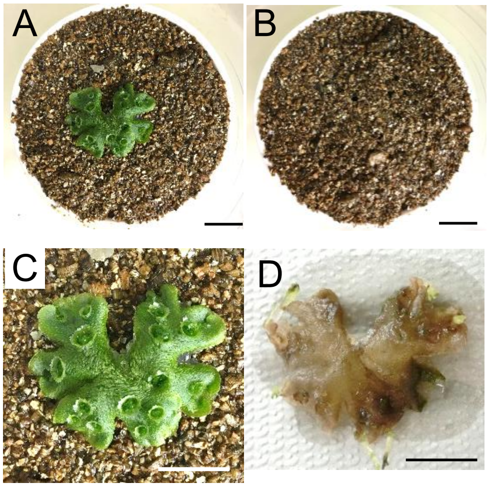

Fig. S2

Narrow structures emerged from thalli buried in the soil. (A) Two-week-old thalli grown under light (B)Thalli were buried in soil at a depth of 1 cm. (C, D) Plants before (C) and after (D) being buried in the soil for 5 weeks. Scale bars = 1cm.

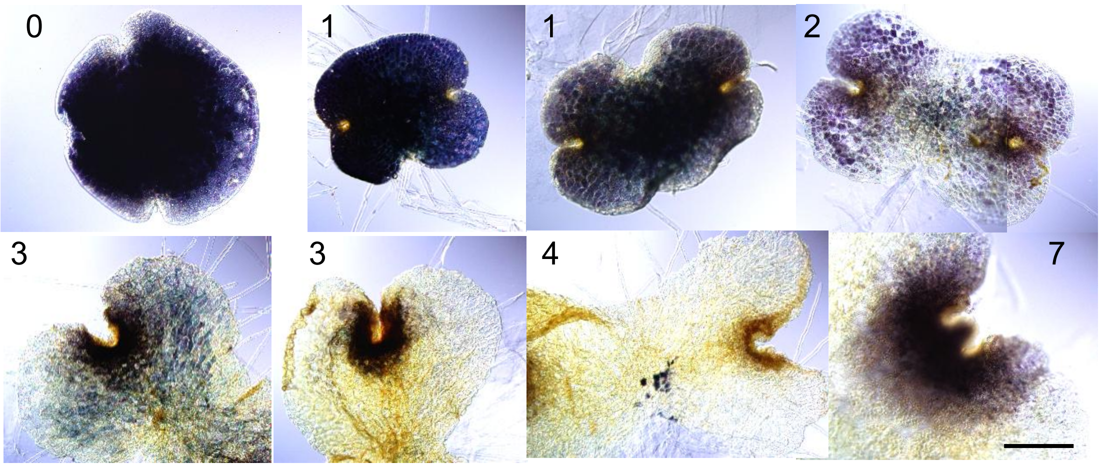

Fig. S3

Young gemmalings lose starch accumulation as they develop. Amyloplasts in light-grown gemmalings were visualized by Lugol's staining. Starch granules stained with Lugol's solution appeared dark blue or purple. The number of days after gemma germination is indicated at the top of each image. Scale bar = 100 µm.

>AtPGM3
TSPIDGQKPG TSGLRKKVKV FNYLENFVQA TFNALTAEKV KGATLVVSGD GRYYSKDAVQ
IIIKMAAANG VRRVWVGKNT LLSTPAVSAV IRERKATGAF ILTASHNPGG PTEDFGIKYN
MENGGPAPES ITDKIYENTK TIKEYPIAPN VDISAVGVTS FGKFDVEVFD PADDYVKLMK
SIFDFEAIRK LLSSPFTFCY DALHGVAGAY AHRIFVEELG AQESALLNCT PKEDFGGGHP
DPNLTYAKEL VARMPEFGAA ADGDADRNMI LGKFFVTPSD SVAIIAANAA IPYFSSGLKG
VARSMPTSAA LDVVAKSLNL KFFEVPTGWK FFGNLMDAGM CSVCGEESFG TGSDHIREKD
GIWAVLAWMS ILAHKNVEDI VRQHWATYGR HYYTRYDYEN VDAGKAKELM EHLVKLQSVA
SVASADEFEY KDPVDGSISK HQGIRYLFED GSRLVFRLSG TGSEGATIRL YIEQYEKDAS
KTGRESQEAL SPLVDLALKL SKMEEFTGRS APTVIT
>MpPGM2
TKPFDGQKPG TSGLRKKVKV FHYLANFVQS SFNALPADRV KGSTIVVSGD GRYYSKDAIQ
IIIRIAAANG VAQVWVGQDG LLSTPAVSGI IRDRKAYGGF ILTASHNAGG PDEDFGIKYN
TENGGPALEA LTDQIYENTK TITTYLIAPD IDIHKIATSS FGPFSVEVFD ATEDYVKMMK
QIFDFEAISR LLARPFTFCY DALHGVAGVY AKRIFLQELG AQESSLLNCT PKEDFGGGHP
DPNLTYAKQL VKTMPEFGAA ADGDADRNMI LGKFFVTPSD SVAIIAANAA IPYFRNGLKG
VARSMPTSAA LDVVAKGLGL RQFEVPTGWK FFGNLMDAGM CSICGEESFG TGSDHIREKD
GIWAVLAWLS ILAYRNVEDI VTLHWGIYGR HYYTRYDYEN VDAGAAKNLM SHLVKLQSVS
GVKSGDEFEY KDPVDGSVSS HQGIRFLFKD GSRLVFRLSG TGSVGATIRL YIEQYQSDKS
KTGADASETL APLVDVALVL SKMEEFTGRS APTVIT
>MpPGM1
TTPIDGQKTG TSGLRKKVKE FNYLANWIQA LFDSLPAEDV KGSTLVLGGD GRYFNKEASQ
IIIKIAAGNG VGKILVGREG IASTPAVSAI IRARKANGGF VMSASHNPGG PKYDWGIKFN
YSSGQPAPES ITDKIYGNTL SIKEIKQAPD VNLSELGVHK FGDFSVEVID PVADYLNLLE
EVFDFDLLKG LLTSKFRFKF DAMHAVTGAY AKPIFVDRLG APEDSIFNGV PLEDFGGGHP
DPNLTYAEEL VKIMPDFGAA SDGDGDRNMI LGNFFITPSD SVAMIAANAA IPYFKTGLKG
LARSMPTSGA LDRVAKELGL PFFETPTGWK FFGNLMDAGK CSVCGEESFG TGSDHVREKD
GIWAVLAWIS IVAYKNVADI AKEHWAKYGR NFFSRYDYEE CESAGANKMV EHLRDIIANY
ELELADDFAY TDPIDGSVAT KQGIRFIFSD GSRIIFRLSG TGSAGATIRI YVEQYEQDTT
KHDLDAQDAL KPLIDIALSV SKLQEFTGRT KPTVIT
>AtPGM2
TSPIDGQKPG TSGLRKKVKV FNYLENFVQA TFNALTTEKV KGATLVVSGD GRYYSEQAIQ
IIVKMAAANG VRRVWVGQNS LLSTPAVSAI IRERKATGAF ILTASHNPGG PTEDFGIKYN
MENGGPAPES ITDKIYENTK TIKEYPIAPR VDISTIGITS FGKFDVEVFD SADDYVKLMK
SIFDFESIKK LLSYPFTFCY DALHGVAGAY AHRIFVEELG APESSLLNCV PKEDFGGGHP
DPNLTYAKEL VARMPEFGAA ADGDADRNMI LGKFFVTPSD SVAIIAANAA IPYFSSGLKG
VARSMPTSAA LDVVAKNLGL KFFEVPTGWK FFGNLMDAGM CSVCGEESFG TGSDHIREKD
GIWAVLAWLS ILAHKNVEDI VRQHWATYGR HYYTRYDYEN VDATAAKELM GLLVKLQSVA
NVASADEFEY KDPVDGSVSK HQGIRYLFED GSRLVFRLSG TGSEGATIRL YIEQYEKDAS
KIGRDSQDAL GPLVDVALKL SKMQEFTGRS SPTVIT
>AtPGM1
TKPIEGQKTG TSGLRKKVKV FNYLANWIQA LFNSLPLEDY KNATLVLGGD GRYFNKEASQ
IIIKIAAGNG VGQILVGKEG ILSTPAVSAV IRKRKANGGF IMSASHNPGG PEYDWGIKFN
YSSGQPAPET ITDKIYGNTL SISEIKVAPD IDLSQVGVTK YGNFSVEVID PVSDYLELME
DVFDFDLIRG LLSRSFGFMF DAMHAVTGAY AKPIFVDNLG AKPDSISNGV PLEDFGHGHP
DPNLTYAKDL VDVMPDFGAA SDGDGDRNMV LGNFFVTPSD SVAIIAANAA IPYFRAGPKG
LARSMPTSGA LDRVAEKLKL PFFEVPTGWK FFGNLMDAGK LSICGEESFG TGSDHIREKD
GIWAVLAWLS ILAHRNVADV VKEYWATYGR NFFSRYDYEE CESEGANKMI EYLREILSNY
VLQFADDFSY TDPVDGSVAS KQGVRFVFTD GSRIIFRLSG TGSAGATVRI YIEQFEPDVS
KHDVDAQIAL KPLIDLALSV SKLKDFTGRE KPTVIT
>HsPGM1
TQAYQDQKPG TSGLRKRVKV FNYAENFIQS IISTVEPAQR QEATLVVGGD GRFYMKEAIQ
LIARIAAANG IGRLVIGQNG ILSTPAVSCI IRKIKAIGGI ILTASHNPGG PNGDFGIKFN
ISNGGPAPEA ITDKIFQISK TIEEYAVCLK VDLGVLGKQQ FKPFTVEIVD SVEAYATMLR
SIFDFSALKE LLSGPLKIRI DAMHGVVGPY VKKILCEELG APANSAVNCV PLEDFGGHHP
DPNLTYAADL VETMHDFGAA FDGDGDRNMI LGKFFVNPSD SVAVIAANII PYFQQTGVRG
FARSMPTSGA LDRVASATKI ALYETPTGWK FFGNLMDASK LSLCGEESFG TGSDHIREKD
GLWAVLAWLS ILATRKVEDI LKDHWQKYGR NFFTRYDYEE VEAEGANKMM KDLEALMFVY
TVEKADNFEY SDPVDGSISR NQGLRLIFTD GSRIVFRLSG TGSAGATIRL YIDSYEKDVA
KINQDPQVML APLISIALKV SQLQERTGRT APTVIT

Table S1 The sequences used in phylogenetic analysis of PGM

>MpAPS1
VLGIILGGGA GTRLYPLTKK RAKPAVPLGA NYRLIDIPVS NCINSNVQKI YVLTQFNSAS
LNRHLSRAYG FVEVLAAQQS PEPNWFQGTA DAVRQYLWLF EENVLEFLVL AGDHLYRMDY
QNFIQAHRDT NADITVAALP EAFGLMKINE KGRIIEFAEK PSMGIYVVSK DAMIKLLRDD
FPANDFGSEV IPYWEDIGTI EAFYNANLGL TKFSFYDRTS PIYTQARFLP PSKMLDADDS
VIGEGCVIKN CKIYHSVVGL RSWIAEGAIV EDALLMGPMG IGRNSIIKRA IIDKNARIGE
NVKIGYFIKS GIVTIIKDAV IPNDTVI
>AtAPS2
VAAIVFGGGS DSELYPLTKT RSKGAIPIAA NYRLIDAVIS NCINSGITKI YAITQFNSTS
LNSHLSKAYR FVEVIAAYQS LEQGWFQGTA DAIRRCLWVF EEPVTEFLVL PGHHLYKMDY
KMLIEDHRRS RADITIVGLS FGFGFMEVDS TNAVTRFTIK GSAGIYVIGR EQMVKLLREC
LISKDLASEI IPYWEDVRSI GAYYRANMES IKYRFYDRQC PLYTMPRCLP PSSMSVAVNS
IIGDGCILDK CVIRGSVVGM RTRIADEVIV EDSIIVGRIG IGEKSRIRRA IVDKNARIGK
NVMIGYVIRE GIIIILRNAV IPNDSIL
>AtAPL3
VAAIILGGGD GAKLFPLTKR AATPAVPVGG CYRMIDIPMS NCINSCINKI FVLTQFNSAS
LNRHLARTYG FVEVLAATQT PGKKWFQGTA DAVRKFLWVF EDNIENIIIL SGDHLYRMNY
MDFVQHHVDS KADITLSCAP SEYGLVNIDR SGRVVHFSEK PSMGVYCFKT EALLKLLTWR
YPSNDFGSEI IPYWEDIGTI KSFYEANIAL VEFEFYDQNT PFYTSPRFLP PTKTEKCRNS
VISHGCFLGE CSIQRSIIGE RSRLDYGVEL QDTLMLGPIG IGRDTKIRKC IIDKNAKIGK
NVVIGFYIRS GITVVVEKAT IKDGTVI
>AtAPL2
VASIILGGGA GTRLFPLTSK RAKPAVPIGG CYRLIDIPMS NCINSGIRKI FILTQFNSFS
LNRHLSRTYG FVEVLAATQT SGKKWFQGTA DAVRQFIWVF EDNVEHVLIL SGDHLYRMDY
MNFVQKHIES NADITVSCLP SDFGLLKIDQ SGKIIQFSEK PSMGVYVFRK EVLLKLLRSS
YPSNDFGSEI IPYWEDIGTI GSFFDANLAL TEFQFYDQKT PFFTSPRFLP PTKVDKCRDS
IVSHGCFLRE CSVQHSIVGI RSRLESGVEL QDTMMMGPVG VGQNTKIKNC IIDKNAKIGK
NVVIGFHIRS GITVVLKNAT IRDGLHI
>MpAPL3
VACMILGGEA GSRLFPLTKR RAKSAVPMGG AYRLIDIQMS NCINSGINKV YVLTQFNSAS
LNRHISRTYG FVEVLAATQT LGKRWFMGTA DAVRRFSWIF DNAVEHVLVL SGDHLYRMNY
MDLVQSHHNS GADITVSCVP SAGGLLRLDH KGRVLSIHDK ASMGLYVFKK DVLIKLLKWI
YPSNDFASEI IPYWQDVGSI ESYFEANLAL TEFEFYDVSN PIYTSPRYLP PTTVDHCRDS
IVSHGCFLRS CSVQHSIIGI RSRIETGVEL KDVVMIGPMG VGEYSKIKRC IIDKNARIGK
NVILGIYIRS GIIIVSENAL IKDGMVI
>AtAPL4
VAAIILGGGN GAKLFPLTMR AATPAVPVGG CYRLIDIPMS NCINSCINKI FVLTQFNSAS
LNRHLARTYG FVEVLAATQT PGKKWFQGTA DAVRKFLWVF EDNIENILIL SGDHLYRMNY
MDFVQSHVDS NADITLSCAP SNFGLVKIDR GGRVIHFSEK PSMGVYCFKT EALLNLLTRQ
YPSNDFGSEV IPYWEDIGTI KTFYEANLAL VEFEFYDPET PFYTSPRFLP PTKAEKCRDS
IISHGCFLRE CSVQRSIIGE RSRLDYGVEL QDTLMLGPIG IGKDTKIRKC IIDKNAKIGK
NVIIGFYIRS GITVIVEKAT IQDGTVI
>E_coli
SVALILAGGR GTRLKDLTNK RAKPAVHFGG KFRIIDFALS NCINSGIRRM GVITQYQSHT
LVQHIQRGWE FVDLLPAQQR MKENWYRGTA DAVTQNLDII RRKAEYVVIL AGDHIYKQDY
SRMLIDHVEK GARCTVACMP SAFGVMAVDE NDKIIEFVEK PSMGIYVFDA DYLYELLEED
DRSHDFGKDL IPYWRDVGTL EAYWKANLDL ASLDMYDRNW PIRTYNESLP PAKFVQDRNS
LVSGGCVISG SVVVQSVLFS RVRVNSFCNI DSAVLLPEVW VGRSCRLRRC VIDRACVIPE
GMVIFYRSEE GIVLVTREML RKLGHKQ
>MpAPL2
VASLILGGGA GTRLFPLTRR RAKPAVPIGG AYRLIDVPMS NCINSGINKV FILTQFNSAS
LNRHLARTYG FVEVLAATQT PGMNWFMGTA DAVRQFTWLF EDGVEHVLIL SGDHLYRMDY
MDFVQKHKDS GADITISCVP SDYGLMKIDH KGQVLYFNEK PSMGIYVFKK EILLKLLRWR
YPANDFGSEI IPYWEDIGTI KSFFDANLAL TEFKFYDVAK PIFTSPRYLP PTKVEKCRDS
IVSHGCFLRD CSVEHSIVGI RSRIESGVEL QDTMMMGPLG VGTNSKIRNC IIDKNSRIGR
NVIIGFYIRS GITVVLKNST IKDGMVI
>AtAPS1
VLGIILGGGA GTRLYPLTKK RAKPAVPLGA NYRLIDIPVS NCLNSNISKI YVLTQFNSAS
LNRHLSRAYG FVEVLAAQQS PEPNWFQGTA DAVRQYLWLF EENVLEYLIL AGDHLYRMDY
EKFIQAHRET DADITVAALP TAFGLMKIDE EGRIIEFAEK PSMGIYVVSR DVMLDLLRNQ
FPANDFGSEV IPYWEDIGTI EAFYNANLGI TKFSFYDRSA PIYTQPRYLP PSKMLDADDS
VIGEGCVIKN CKIHHSVVGL RSCISEGAII EDSLLMGPIG IGKNSHIKRA IIDKNARIGD
NVKIGYFIKS GIVTVIKDAL IPTGTVI
>AtAPL1
VASIILGGGA GTRLFPLTKR RAKPAVPIGG AYRLIDVPMS NCINSGINKV YILTQYNSAS
LNRHLARAYG YVEVLAATQT PGKRWFQGTA DAVRQFHWLF EDDIEDVLIL SGDHLYRMDY
MDFIQDHRQS GADISISCIP SDFGLMKIDD KGRVISFSEK PSMGVYVFKK EILLNLLRWR
FPANDFGSEI IPYWEDIGTI RSFFEANLAL TEFSFYDAAK PIYTSRRNLP PSKIDNSKDS
IISHGSFLTN CLIEHSIVGI RSRVGSNVQL KDTVMLGPIG IGENTKIQEC IIDKNARVGK
NVIIGFYIRS GITVILKNSV IKDGVVI
>MpAPL1
VVSLILGGGA GTRLFPLTKR RAKPAVPIGG GYRLIDVPMS NCINSGINKV FILTQFNSAS
LNRHLARTYG FVEVLAATQT PGKEWFQGTA DAVRQYLWLF EDNLEDVLIL SGDHLYRMDY
MDFVEKHRNS GADITISCVP SDYGLMKIDD TGRVLYFSEK PSMGIYVFKK EILQKLLRWR
YPANDFGSEI IPYWEDIGTI KSFFDANLGL TAFSFYDAVK PIFTSPRYLP PSKIEKCRDS
IISHGCFLRD CSIKHSIVGI RSQMASGSAL KDTMMLGPIG VGANCKISNC IIDKNARIGS
NVVIGYYIRS GIVVILKNST IAPGTVI

Table S2 The sequences used in phylogenetic analysis of APL

Primer name	Sequences
MpApS1_Fw	CACCATGGCTGGTGTTGCGACAG
MpApS1_Rv	GATGACGGTGTCGTTGGGAA
MpAPS1_gDNA_Fw	CTCGAGTTGCCTGACGCCCGAGGC
MpAPS1_gDNA_Rv	AAACGCCTCGGGCGTCAGGCAACT
MpAPS1_GT1_Fw	CTTACTGCTGCTCGCTTACCT
MpAPS1_seq1_Fw	GCACAACCGCTGAGGTCG
MpPGM1_gDNA_Fw	CTCGGCATGGCGAGACCGCGCGCC
MpPGM1_gDNA_Rv	AAACGGCGCGCGGTCTCGCCATGC
MpPGM1_GT1_Fw	AAGTGGACCGCCCACGGCAT
MpPGM1_GT2_Rv	CCACTAGTTCCAGTCTTCTGGC
MpPGM1_seq1_Fw	GCGCGCACAGATGCGAAT

Table S3 Primers used in this study

	The length of narrow thalli (Mean ± SE cm))
Tak-1 (1G)	1.13 ± 0.06
Clinostat	1.20 ± 0.05

No significant difference (N=58, Student's t-test P=0.069)

Table S4

The length of narrow structures treated with and without clinorotation

	The length of narrow thalli (Mean ± SE cm)
Tak-1	0.80 ± 0.09
Mp*pgm1-1*^{ge}	0.78 ± 0.05
Mp*aps1-1*^{ge}	0.96 ± 0.05
Mp*aps1-2*^{ge}	0.73 ± 0.07

No significant differences (N=24, One-way ANOVA, P=0.08).

Table S5

The length of narrow structures in WT and starchless mutants
