# Supplementary figures and images for: Amyloplasts are necessary for full gravitropism in thallus of Marchantia polymorpha
Source: J Exp Bot. 2025 Aug 19;76(22):6741–57. doi: 10.1093/jxb/eraf375 (PMC12675261; doi:10.1093/jxb/eraf375)

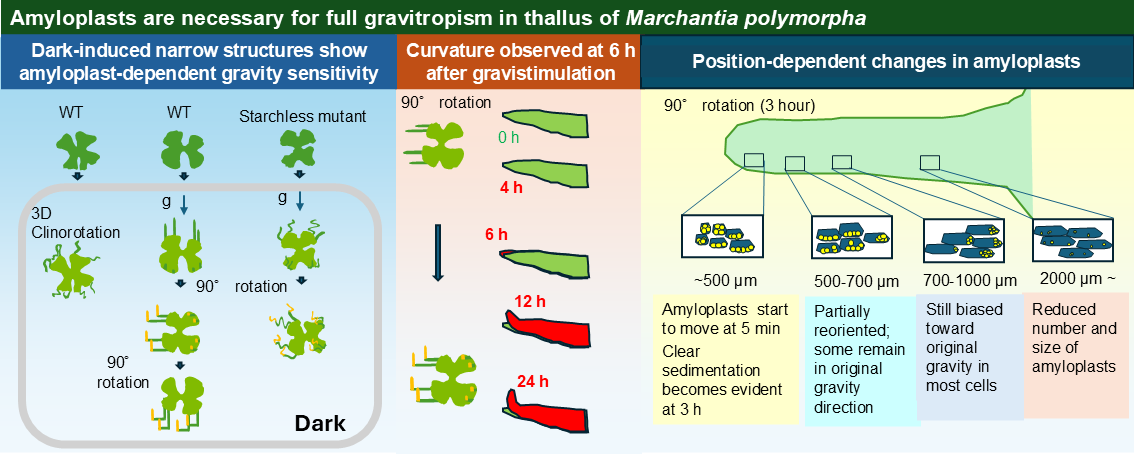

Supplement: eraf375_Supplementary_Data [file eraf375_supplementary_data.zip › jexbot314494-file002.tif]
